# Regional variability and genotypic and pharmacodynamic effects on PrP concentration in the CNS

**DOI:** 10.1172/jci.insight.156532

**Published:** 2022-03-22

**Authors:** Meredith A. Mortberg, Hien T. Zhao, Andrew G. Reidenbach, Juliana E. Gentile, Eric Kuhn, Jill O’Moore, Patrick M. Dooley, Theresa R. Connors, Curt Mazur, Shona W. Allen, Bianca A. Trombetta, Alison McManus, Matthew R. Moore, Jiewu Liu, Deborah E. Cabin, Holly B. Kordasiewicz, Joel Mathews, Steven E. Arnold, Sonia M. Vallabh, Eric Vallabh Minikel

**Affiliations:** 1 Stanley Center for Psychiatric Research, Broad Institute of MIT and Harvard, Cambridge, Massachusetts, USA.; 2 Ionis Pharmaceuticals Inc., Carlsbad, California, USA.; 3 Proteomics Platform, Broad Institute of Massachusetts Institute of Technology and Harvard, Cambridge, Massachusetts, USA.; 4 McLaughlin Research Institute, Great Falls, Montana, USA.; 5 Massachusetts Alzheimer’s Disease Research Center, and; 6 McCance Center for Brain Health, Massachusetts General Hospital, Boston, Massachusetts, USA.; 7 Department of Neurology, Massachusetts General Hospital and Harvard Medical School, Boston, Massachusetts, USA.; 8 Bioagilytix, Boston, Massachusetts, USA.; 9 Prion Alliance, Cambridge, Massachusetts, USA.

**Keywords:** Neuroscience, Therapeutics, Neurodegeneration, Prions

## Abstract

Prion protein (PrP) concentration controls the kinetics of prion replication and is a genetically and pharmacologically validated therapeutic target for prion disease. In order to evaluate PrP concentration as a pharmacodynamic biomarker and assess its contribution to known prion disease risk factors, we developed and validated a plate-based immunoassay reactive for PrP across 6 species of interest and applicable to brain and cerebrospinal fluid (CSF). PrP concentration varied dramatically across different brain regions in mice, cynomolgus macaques, and humans. PrP expression did not appear to contribute to the known risk factors of age, sex, or common *PRNP* genetic variants. CSF PrP was lowered in the presence of rare pathogenic *PRNP* variants, with heterozygous carriers of P102L displaying 55%, and D178N just 31%, of the CSF PrP concentration of mutation-negative controls. In rodents, pharmacologic reduction of brain *Prnp* RNA was reflected in brain parenchyma PrP and, in turn in CSF PrP, validating CSF as a sampling compartment for the effect of PrP-lowering therapy. Our findings support the use of CSF PrP as a pharmacodynamic biomarker for PrP-lowering drugs and suggest that relative reduction from individual baseline CSF PrP concentration may be an appropriate marker for target engagement.

## Graphical abstract

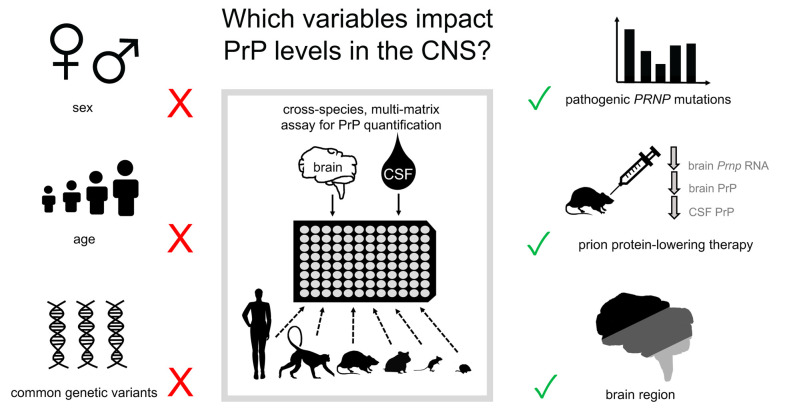

## Introduction

Prion disease is a fatal neurodegenerative disease caused by misfolding of the prion protein (PrP) leading to a gain of toxic function (1). Lowering PrP expression in the brain is a potential therapeutic approach thoroughly underpinned by genetic proofs of concept (2, 3). Antisense oligonucleotides (ASOs) that lower PrP extend survival by up to 3-fold in prion-infected mice (4–6), supporting the further development of PrP-lowering drugs. This motivates a need to accurately measure the degree to which PrP has been lowered upon drug treatment, across a variety of species and matrices. Such quantification of target engagement — a drug’s impact on its intended molecular target — is critical throughout the life cycle of any drug development program, from therapeutic candidate screening and lead optimization, to in vitro and in vivo pharmacology studies in animals, to dose selection and confirmation of drug activity in human clinical trials. In prion disease, quantification of PrP may play an even larger role: lowering of cerebrospinal fluid (CSF) PrP in presymptomatic individuals at high risk for genetic prion disease could be employed as a surrogate biomarker endpoint in support of provisional drug approval (3).

In previous studies, PrP in human CSF was quantified using a commercially available ELISA assay specific to human PrP (7–11), as well as a multiple reaction monitoring (MRM) targeted mass spectrometry assay (12). PrP is highly abundant in human CSF (12), on the order of tens or hundreds of nanograms per milliliter. CSF PrP concentration paradoxically decreases in symptomatic prion disease (7–10, 12, 13) amidst a toxic buildup of PrP in the brain, but no decline in CSF PrP was observed in presymptomatic mutation carriers (11). PrP sticks to plastic, and is thus exquisitely sensitive to preanalytical variables, but with uniform sample handling and addition of detergent, CSF PrP can be reliably quantified (10), with a test-retest mean coefficient of variation (CV) of only 7% in serial samples collected from the same individuals over more than a year (11). These findings support the use of CSF PrP as a pharmacodynamic biomarker to measure target engagement in presymptomatic individuals.

Despite this strong foundation, the development path for PrP-lowering therapeutics faces several outstanding practical needs, including improved measurement tools both to track treatment response and to better address unresolved biological questions about disease pathophysiology. Drug development activities will be facilitated by establishment of an inexpensive, easy-to-implement assay capable of measuring PrP both in humans and across relevant animal species, in both the brain and CSF. Advanced age, male sex, and both rare and common *PRNP* genetic variants are risk factors for prion disease (14, 15), and it is unknown whether differences in PrP expression contribute to any of these factors. Regional differences in brain PrP expression (16–18) might interact with drug distribution patterns in the brain (19) to influence biomarker and clinical outcomes in future trials. Expectations that pharmacologic lowering of PrP RNA in the brain should be reflected in brain PrP and consequently in CSF PrP should be experimentally demonstrated in animals to validate the use of CSF as a sampling compartment. Here, we develop a new cross-species PrP ELISA assay, assess its performance characteristics, and deploy it across a range of animal and human samples to address the above questions.

## Results

### Cross-species ELISA assay.

After screening 4 commercially available anti-PrP monoclonal antibodies in pairs for sensitivity and cross-reactivity (Supplemental Figure 1; supplemental material available online with this article; https://doi.org/10.1172/jci.insight.156532DS1), we developed a final assay protocol (Supplemental Appendices 1 and 2) using monoclonals EP1802Y for capture and 8H4 for detection, with C-terminal epitopes mapped approximately to residues 218–227 and 182–196 (human codon numbering) (20–22). The assay possesses dynamic range from 0.05 to 5.0 ng/mL, exhibits linearity for endogenous PrP in mouse brain homogenate, and meets FDA criteria for bioanalytical method validation (23) (Supplemental Tables 1 and 2), except for elevated interplate variability near the lower limit of quantification (Supplemental Table 1). In brain homogenate, quantification of PrP required 0.2% CHAPS to fully solubilize PrP (Supplemental Figure 1), and also benefited from minimization of time spent above freezing (Supplemental Table 2), and plating at uniform dilution (Supplemental Figure 2). The assay is applicable to both brain and CSF and is equally reactive with human, cynomolgus macaque, mouse, rat, and bank vole PrP, with slightly reduced reactivity for Syrian hamster PrP (Supplemental Figures 2–4, and Supplemental Table 3).

### Regional distribution of brain PrP.

**Figure 1:**
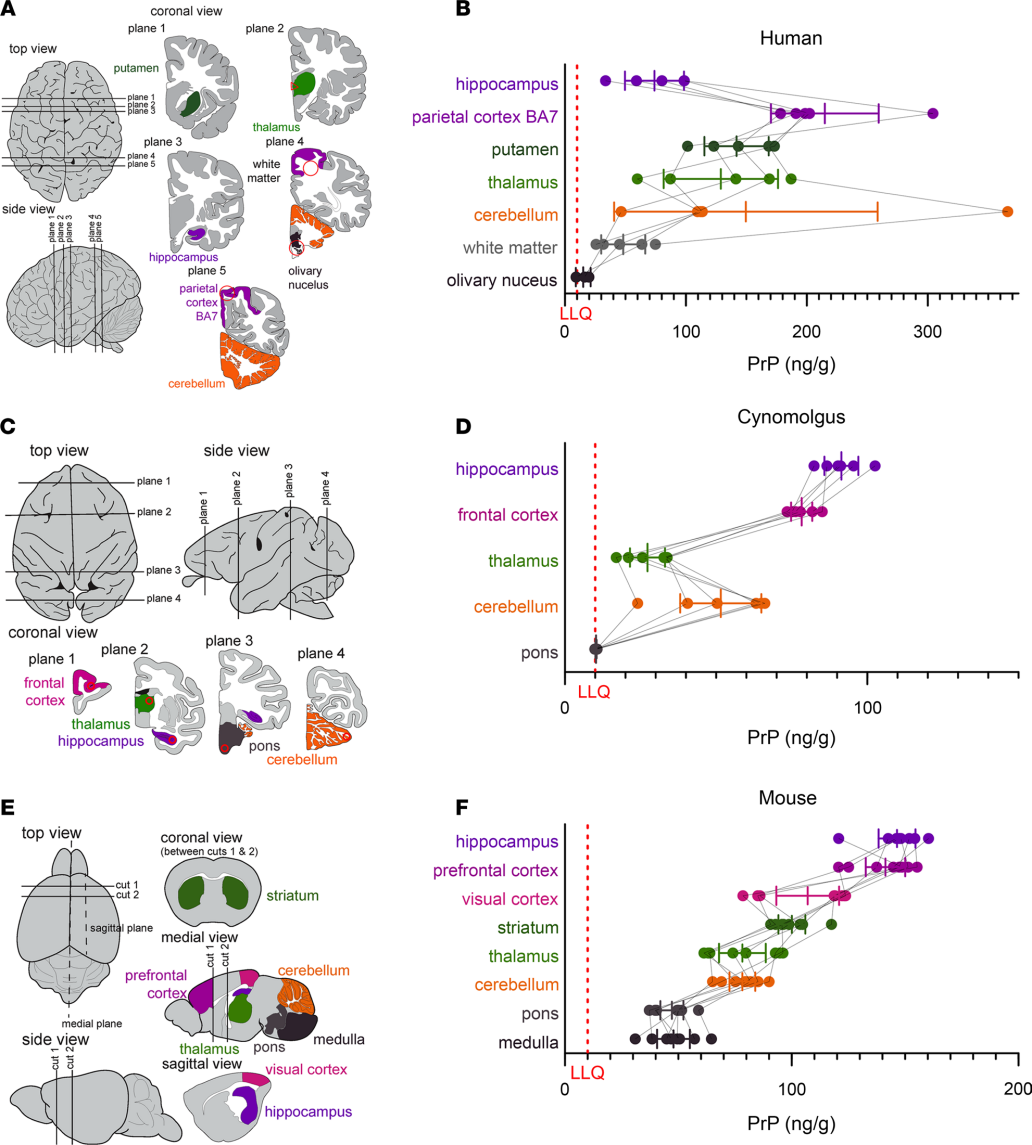
Regional distribution of brain PrP. (**A**, **C**, and **E**) Diagrams of brain regions examined in humans, cynomolgus macaques, and mice, respectively, and (**B**, **D**, and **F**) PrP concentrations in *n* = 5 human, *n* = 6 macaque, and *n* = 8 mouse brains. Thin lines connect regions from the same individual. Bars indicate mean and 95% confidence interval of the mean. Red dashed lines indicate lower limit of quantification (LLQ). Brain diagrams were traced from Allen Brain Atlas images (51, 52).

We previously observed an approximately 8-fold difference in PrP concentrations among *n* = 28 human brain samples (10), which could have reflected one or more of the following: brain region differences, interindividual differences, effects of agonal state or postmortem interval, and/or preanalytical variation due to incomplete solubilization of PrP in the 0.03% CHAPS buffer used at that time. We therefore obtained a new set of 6–7 matched brain regions from each of 5 control individuals, and homogenized them in 0.2% CHAPS for analysis by cross-species PrP ELISA. We identified considerable regional differences (*P* = 0.0007, Type I ANOVA), with PrP almost 10 times higher in parietal cortex (BA7) than in olivary nuclei (Figure 1, A and B). Analogous regional disparities were observed in cynomolgus macaques (*P* < 1 × 10^–10^, type I ANOVA, Figure 1, C and D) and mice (*P* < 1 × 10^–10^, type I ANOVA, Figure 1, E and F). Across all 3 species, PrP concentrations were higher in cortex than in subcortex and cerebellum, and were lowest in brainstem, although humans displayed the highest interindividual variability and a low PrP concentration in hippocampus in contrast to mouse and macaque (see Discussion).

### Assessment of sex and age effects on PrP expression.

**Figure 2:**
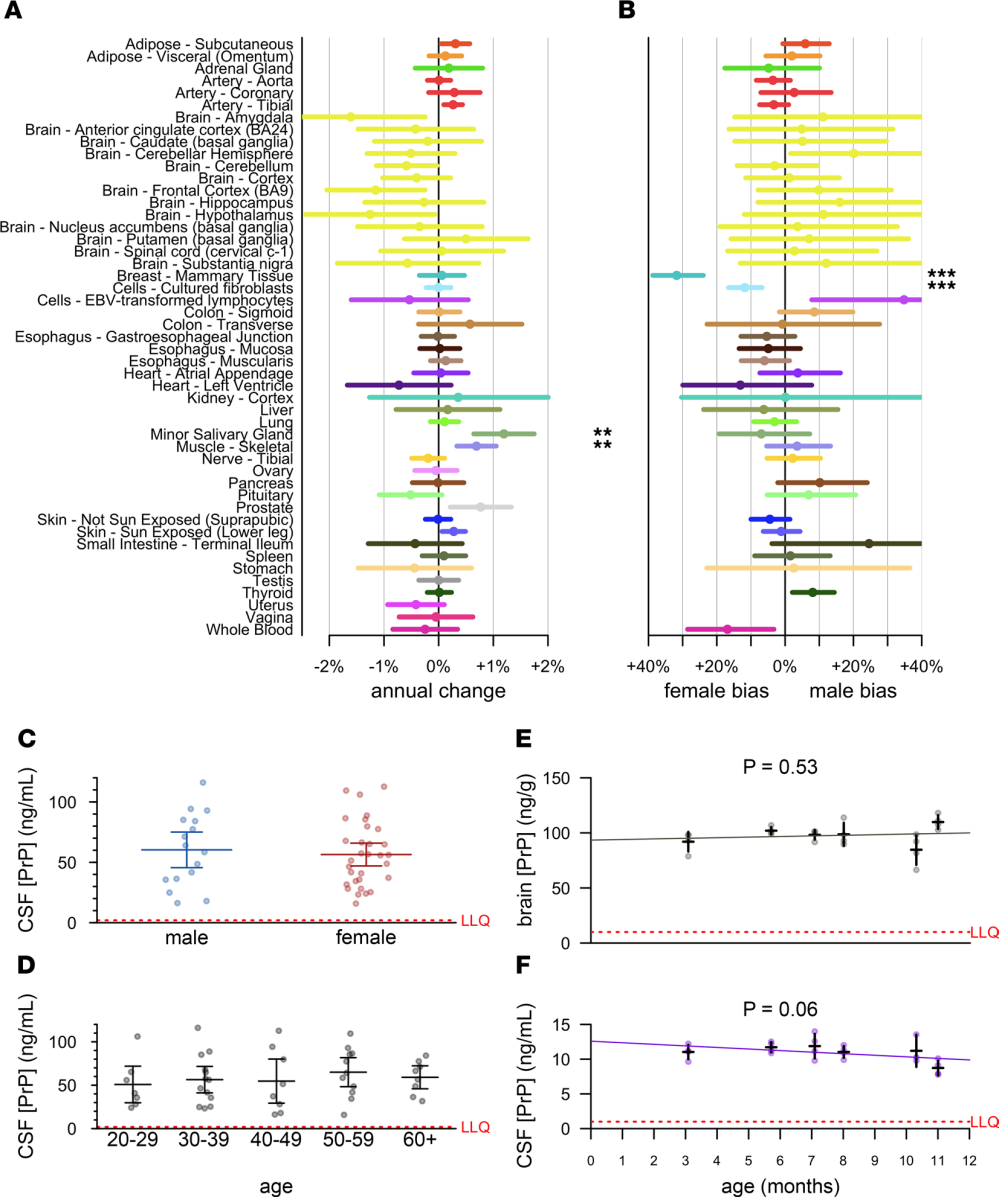
Lack of evidence for sex or age effects in PrP expression. (**A** and **B**) Analysis of publicly available GTEx v8 data. Log-linear models [log(tpm) ~ age + hardy + sex; see Results] were fit for each tissue, and the mean annual change (dots) was calculated as exp(β_age_)-1 and exp(β_sex_), with 95% confidence intervals (line segments) given by 1.96 standard errors of the mean. After Bonferroni correction for *n* = 49 tests (**A**) or *n* = 44 tests (**B**), symbols indicate *P < 0.05, **P < 0.01, ***P < 0.001. (**C** and **D**) CSF PrP concentrations averaged across all available CSF samples for *n* = 47 MGH study participants stratified by sex (**C**) or age (**D**). Bars indicate mean and 95% confidence interval of the mean. (**E** and **F**) Brain (**E**) and CSF (**F**) concentrations of PrP for cohorts of *n* = 4 male Sprague-Dawley rats aged 3–11 months. Red dashed lines indicate lower limit of quantification (LLQ).

We first analyzed *PRNP* RNA levels in the GTEx v8 dataset (24). After controlling for cause of death (4-point Hardy scale), which is confounded with sex and with decade of life (*P* < 1 × 10^–10^ for both, χ^2^ test), and correcting for multiple testing, only minor salivary gland and skeletal muscle showed any evidence of age-dependent expression (higher with age, Figure 2A), and only mammary tissue and cultured fibroblasts showed evidence of sex-biased expression (higher in females, Figure 2B). We found no evidence that *PRNP* RNA expression in any brain region (yellow, Figure 2, A and B) correlated with age or sex. PrP protein expression might nevertheless change in brain parenchyma due to changes in translation or degradation rates; however, considering differences in PrP concentration across brain regions found here (Figure 1) and by others (16–18), and the potential impact of preanalytical variables (Supplemental Table 2), we were unable to identify a sample set of human brains suitable for querying age differences. We therefore confined our subsequent analyses to human CSF and to rat brain and CSF.

We measured PrP in CSF from *n* = 47 individuals (healthy asymptomatic *PRNP* mutation carriers and non-carrier controls) from our cohort study at Massachusetts General Hospital (MGH, Boston, Massachusetts, USA) (11). Exquisite uniformity of CSF handling plus early addition of 0.03% CHAPS minimized preanalytical confounders in this cohort. Among the *n* = 36 of these individuals who had more than 1 serial sample (range: 2–5 lumbar punctures performed over a period of up to 3.5 years), CSF PrP measured in cross-species ELISA exhibited tight test-retest reliability (mean CV = 11.1%). We therefore focused on each individual’s mean CSF PrP value observed across all visits. We found no evidence for CSF PrP association with sex (*P* = 0.81, Kolmogorov-Smirnov test, Figure 2C), nor age (*P* = 0.28, Spearman correlation, Figure 2D). In male rats aged 3–11 months, PrP concentrations in neither brain (Figure 2E) nor CSF (Figure 2F) changed with age.

### Genotypic effects on human CSF PrP concentration.

**Figure 3:**
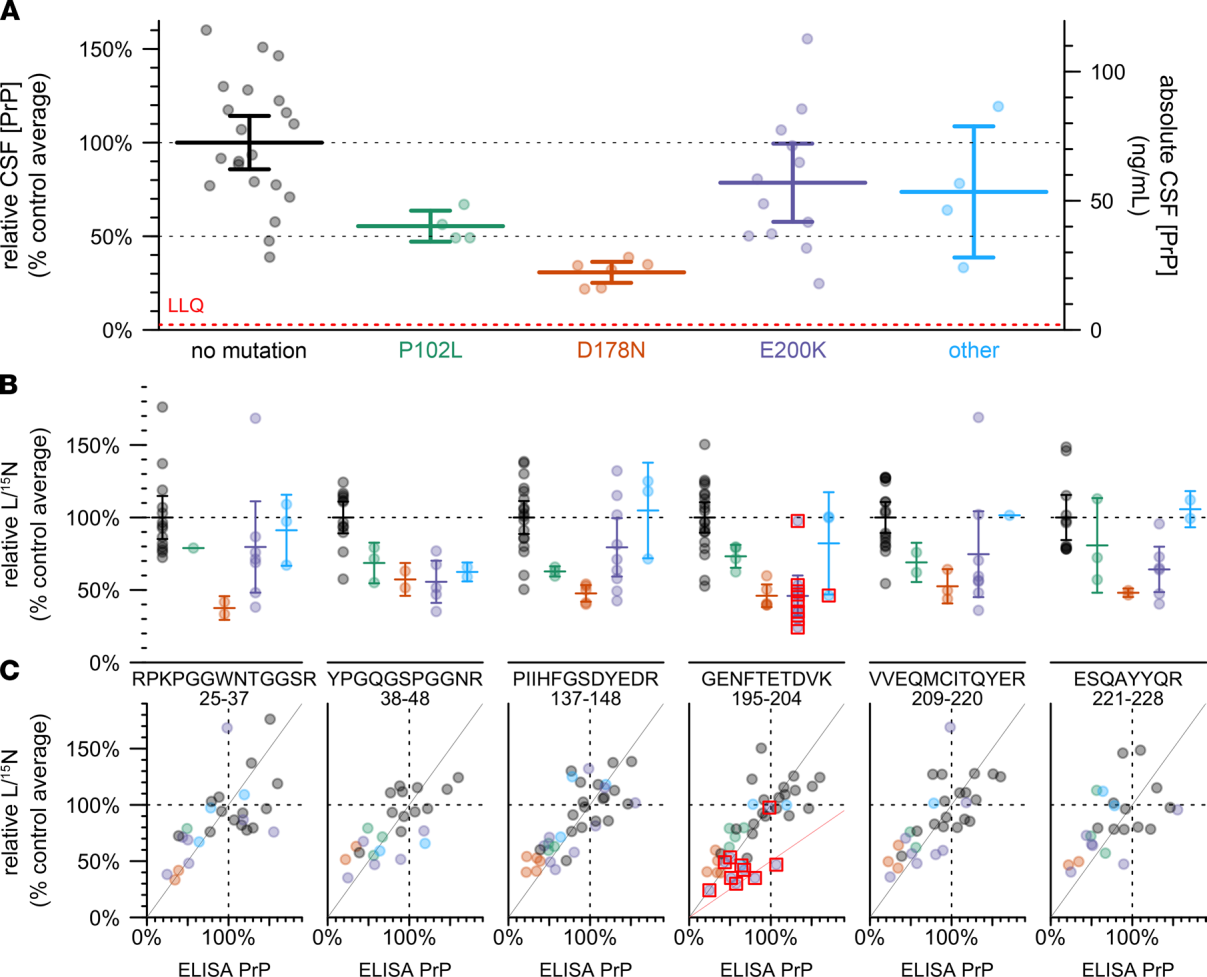
Effect of PRNP mutation on CSF PrP concentration. (**A**) CSF PrP concentrations measured by cross-species ELISA, averaged across all available CSF samples for each of *n* = 47 MGH study participants, normalized to the mean of non-mutation carrier controls. Bars indicate mean and 95% confidence interval of the mean. Red dashed line indicates lower limit of quantification (LLQ). This sample set includes *n* = 29 individuals for which CSF PrP concentrations determined by BetaPrion ELISA were previously reported (11). (**B**) The same samples analyzed by the PrP MRM assay, peptides arranged from N-terminal (left) to C-terminal (right). Peptide sequences and residue numbers are noted beneath each plot. Because observations with technical replicate CVs greater than 15%, were removed, the number of samples differs for each panel. Bars indicate mean and 95% confidence interval of the mean. (**C**) Correlation between ELISA results from (**A**) (*x* axis) and MRM results from (**B**) (*y* axis), with lines indicating a diagonal with slope = 1 (gray) and 0.5 (pink, GENFTETDVK only). In (**B** and **C**), red boxes indicate individuals whose mutation abolishes the tryptic peptide being monitored in that plot.

In *n* = 47 cohort study participants with at least 1 CSF sample available, we examined genotypic differences in CSF PrP by comparing mean CSF PrP levels averaged, for each individual, across all study visits (Figure 3A). Compared to mutation-negative controls (*n* = 21), CSF PrP was lower for carriers of P102L (55%, *P* = 0.0055, Kolmogorov-Smirnov test, *n* = 4) and D178N (31%, *P* = 6.7 × 10^–6^, Kolmogorov-Smirnov test, *n* = 6); the trend was preserved but non-significant for E200K (78%, *P* = 0.23, Kolmogorov-Smirnov test, *n* = 12).

ELISA relies on the presence of 2 intact epitopes on the same protein, so non-reactivity of 1 of our antibodies for 1 of these mutations could give rise to artifactual genotypic differences. The 8H4 epitope has been mapped to a region adjacent to D178N and E200K (20, 21), and some PrP mutations are reported to affect interdomain interactions (25) and could therefore alter the accessibility even of distal epitopes (21). We therefore employed a targeted mass spectrometry method (12) using stable isotope–labeled amino acids and MRM, on CSF, to measure 6 tryptic peptides spanning the N- to C-termini of PrP. Individuals with the E200K, P102L, and particularly D178N mutations had lower mean levels of all 6 peptides, compared to mutation-negative controls (Figure 3B). Indeed, across individual samples, those that were low in ELISA were low in MRM and those that were high in ELISA were high in MRM, with samples clustering along the diagonal with a slope equal to 1 (gray line, Figure 3C). For peptide GENFTETDVK, those individuals whose mutations disrupt this peptide (mostly E200K individuals; red boxes, Figure 3C) clustered closer to a line with slope equal to 0.5 (pink line, Figure 3C), consistent with non-detection of this peptide from the mutant allele. Overall, the fact that each peptide observed in MRM replicated the ELISA result confirmed that CSF PrP was genuinely reduced in a genotype-dependent manner in individuals with certain pathogenic *PRNP* mutations.

We also examined 2 common variants in *PRNP*: M129V (rs1799990) and a non-coding variant 72 kb upstream of *PRNP* (rs17327121) implicated as the lead variant for an expression quantitative trait locus (eQTL) in cerebellum (24). Neither was significantly correlated with CSF PrP in our samples (Supplemental Figure 5).

### Pharmacodynamic effect of PrP RNA-targeting therapy in rodents.

*Prnp*-targeting ASOs that extend survival in vivo do so by lowering *Prnp* RNA (5, 6, 26). This reduction in *Prnp* RNA is expected to lead to lowering of brain parenchyma PrP, and in turn to reduction of PrP released into CSF, but the relationships among these variables have not yet been quantitatively investigated.

**Figure 4:**
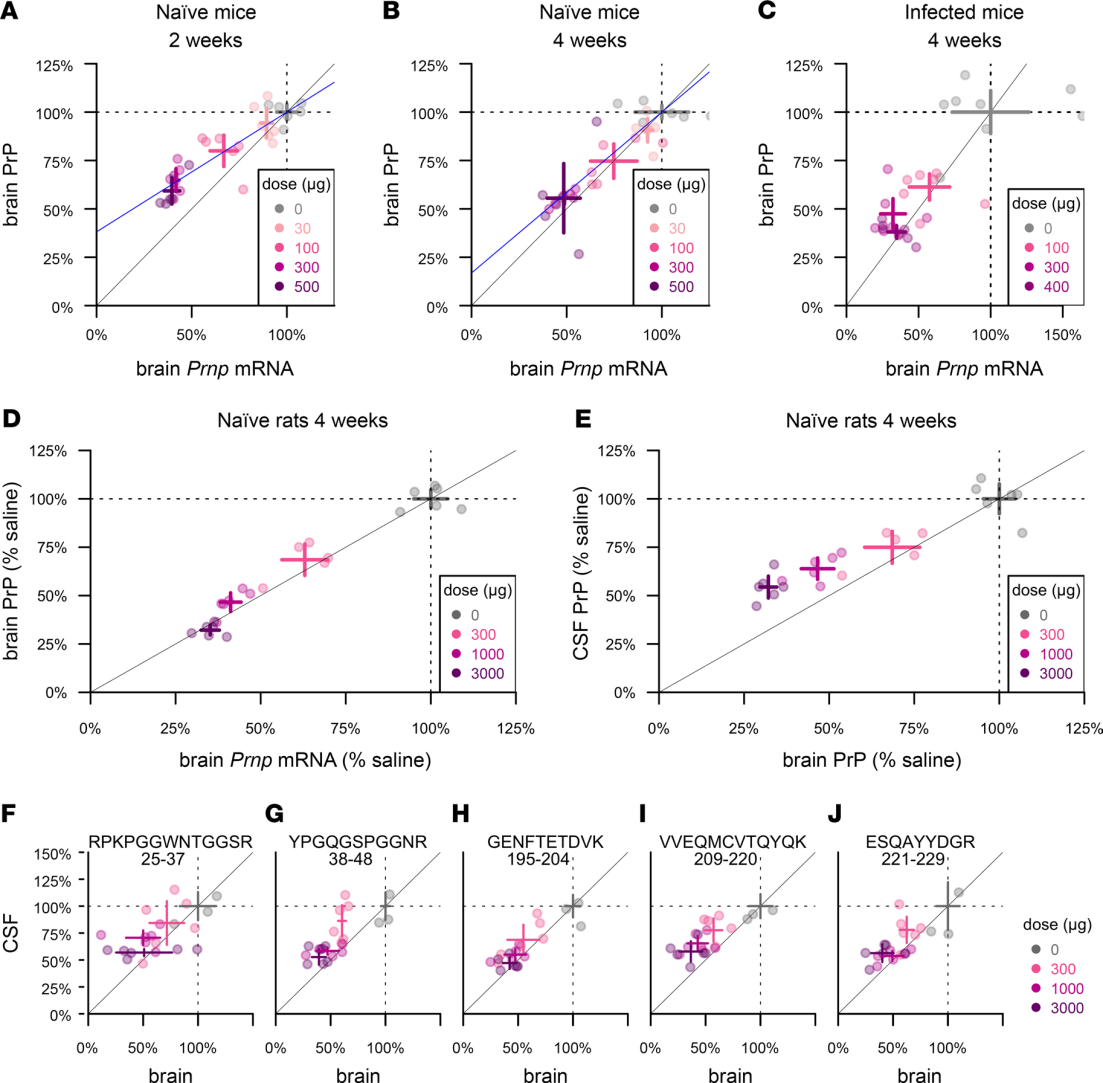
Pharmacodynamic effect of PrP RNA-targeting therapy. (**A** and **B**) Whole-hemisphere RNA (*x* axis) versus PrP (*y* axis), reduction measured by ELISA in groups of *n* = 6 naive mice at 2 weeks (**A**) and 4 weeks (**B**) after dosing. Blue lines represent linear regression best fits with the (1,1) coordinate fixed. (**C**) RNA from the lateral half of 1 hemisphere (*x* axis) versus PrP from the medial half of the same hemisphere (*y* axis), reduction measured by ELISA in groups of *n* = 6 RML prion–infected mice dosed at 60 dpi and harvested at 4 weeks after dosing. (**D**) Whole-hemisphere RNA (*x* axis) versus PrP (*y* axis), reduction measured by ELISA in groups of *n* = 6 naive rats harvested at 4 weeks after dosing. (**E**) Whole hemisphere PrP (*x* axis) reduction versus CSF PrP (*y* axis) in the same rats. (**F–J**) CSF and brain samples from (**E**) analyzed by MRM, with the 5 rat PrP peptides arranged from N-terminal (**F**) to C-terminal (**J**). Peptide sequences and residue numbers are noted above each plot. In every panel, crosshairs represent the mean and 95% confidence interval of the mean on both dimensions.

We first sought to understand the relationship between whole-brain *Prnp* RNA and protein levels in mice using ASO 6, a tool compound previously shown to extend survival of prion-infected mice (6). At 2 and 4 weeks after dosing in naive animals, active ASO 6 dose-dependently suppressed whole hemisphere PrP (Figure 4, A and B). Protein suppression was weaker than RNA suppression at 2 weeks, with each 1% reduction in *Prnp* RNA corresponding to just a 0.62% reduction in PrP (Figure 4A). The 2 measures were in closer agreement by 4 weeks, with each 1% RNA reduction corresponding to 0.83% PrP reduction (Figure 4B). We observed comparable target engagement and close correspondence between RNA and protein levels in RML prion–infected animals treated at 60 days postinoculation (dpi) and harvested 4 weeks after dosing at 88 dpi (Figure 4C).

Because CSF PrP is more sensitive to plastic adsorption when handled in very small volumes (10), it would be challenging to measure CSF PrP reduction upon drug treatment in mice. We therefore examined the relationships among *Prnp* mRNA, brain PrP, and CSF PrP in rats (Figure 4, D and E). At 4 weeks after dosing, whole hemisphere PrP was dose-dependently suppressed in proportion to whole hemisphere *Prnp* mRNA (Figure 4D). The reduction in brain PrP was in turn reflected in CSF PrP, although CSF PrP reduction slightly underestimated the depth of target engagement in brain parenchyma, with each 1% reduction in CSF PrP knockdown corresponding to a 1.4% reduction in brain PrP (Figure 4E). The relationship between PrP knockdown in brain and in CSF was reproduced by MRM, and did not differ significantly among the 5 peptides examined (*P* = 0.14, ANCOVA; Figure 4, F–J).

## Discussion

Given the pivotal role of PrP in prion biology, it is reasonable to ask whether any known risk factors for prion disease, including age, sex, and genotype, are mediated by differences in PrP expression. Two previous studies observed suggestive associations between CSF PrP concentration and age (10, 13), but only in historical cohorts where preanalytical variables were not well-controlled and/or samples were not well-matched on other variables. One previous study indicated that PrP expression on peripheral leukocytes rose with age (27), but no such change in brain has been reported beyond the first few weeks of life (17, 28). If PrP expression in brain rose with age, this could potentially explain the mid- to late-life onset of most prion disease, even in the lifelong presence of a pathogenic mutation (29, 30). We found no evidence, however, that human brain *PRNP* RNA expression, PrP concentration in human CSF, or PrP in rat brain and CSF change with age. If PrP expression were indeed sex-biased, this could potentially explain the reportedly higher incidence of prion disease in men (14) (risk ratio = 1.2). We found no evidence, however, from publicly available RNA data, nor from our own analyses of human CSF, to support a sex difference in PrP expression. Common variants in *PRNP* are associated with prion disease risk, but this risk exhibits no obvious connection to *PRNP* expression (31). The common variant M129V affects the risk and histopathological subtype of sporadic and acquired prion disease as well as disease duration in genetic prion disease (15, 30), but while it is the lead SNP for a peripheral tissue eQTL, it is not an eQTL in human brain (24). We found no evidence that M129V affects CSF PrP. The lead variant for a reported cerebellar eQTL 72 kb upstream of *PRNP* (24), which is not known to be associated to prion disease risk (31), likewise showed no evidence of influencing CSF PrP. All of the above analyses are underpowered for small effect sizes, but use of larger historical CSF cohorts to interrogate these questions would be complicated by the uncontrolled preanalytical variability present in such samples (10). While our findings do not rule out sex, age, or common variant effects on PrP expression, they may suggest that any such effects are too small to be major confounders in a clinical trial enrolling tens of individuals.

CSF PrP concentrations are dramatically lower, however, in individuals with some pathogenic *PRNP* mutations. The number of individuals examined here remains small, and includes samples reported previously (11). Nevertheless, the difference is large and unambiguously statistically significant, and this finding replicates across 2 ELISA assays (11) and 6 peptides monitored by MRM, ruling out an immunoreactivity artifact. This genotypic difference has been maintained over years of follow-up and in the absence of detectable prodromal pathological changes (11), which appear to occur only very shortly before onset in prion disease (32). We therefore conclude that these mutations lead to constitutively lower concentrations of PrP in CSF. In principle, this could arise from any combination of the following: reduced translation, faster catabolism, or reduced shedding of PrP into CSF. Studies of D178N in animal and cell culture models favor faster catabolism, perhaps secondary to impaired trafficking, and thus lower steady state levels in parenchyma for this mutation (33–36). CSF PrP in D178N carriers averaged just 31% that of non-carrier controls. That this number is less than 50%, despite all of our carriers being heterozygotes, raises the possibility that the presence of the mutation suppresses the expression or shedding of the wild-type protein, or shortens its half-life, in *trans*. This possibility, and the mechanism that might govern it, warrant further study.

That CSF PrP differs by genotype prompts consideration of the basis — relative or absolute — on which target engagement should be assessed in clinical trials of PrP-lowering drugs. Clinical trials of ASOs for other targets have generally reported relative reductions in target biomarkers — percentage declines from individual baselines (37, 38). There also exists, however, precedent for therapies being dosed to target an absolute level of a response biomarker. The best predictor of efficacy for the antibody omalizumab in severe asthma is the patient’s free IgE after treatment, with the goal of reducing levels to below 25 ng/mL (39). Thus, some may ask whether PrP-lowering therapies should be dosed to keep CSF PrP below some absolute ng/mL level. D178N is highly penetrant (40) and exhibits earlier average onset than E200K (30) despite reduced basal levels of CSF PrP. This argues that, although CSF PrP appears usable as a therapeutic response marker in prion disease, absolute levels of this analyte may not hold significance that generalizes across individuals. Thus, a single absolute threshold would likely not serve as an appropriate treatment goal.

A proposed pathway (3) whereby a PrP-lowering drug could receive provisional approval based on lowering of CSF PrP relies crucially on lowering PrP in brain being reflected in CSF. Here we empirically validated this link in rats, and showed that the response is uniform across tryptic peptides spanning the length of PrP. Thus, CSF PrP appears to be 1 analyte, with multiple different measurement methods all reflecting the concentration of the disease-relevant protein.

We found that PrP concentration varies dramatically across different brain regions in humans, in monkeys, and in mice. The pattern was generally consistent across species and agrees with previous reports in rodents (17, 18). Human brain samples exhibited much higher interindividual variability than monkeys or mice, however, and only modest PrP concentrations were detected in human hippocampal samples, whereas this region had the single highest PrP concentration in both mice and monkeys. These observations might reflect the size of the human brain. For example, whereas we analyzed whole mouse hippocampi, in humans we examined an approximately 160 mg medial slice of CA1-4, posterior to the globus pallidus. If PrP expression is highly enriched in certain subregional structures (17, 18), then slight dissection differences could yield varying results. Nonetheless, our findings across species showed that PrP expression exhibits genuinely large variability across regions, and this should be accounted for when modeling which regions contribute to pharmacodynamic signal in CSF.

Our findings also bear on the timescale on which the pharmacodynamic effect of PrP-lowering therapies can be observed. PrP’s half-life in the mouse brain was estimated at just 18 hours in a conditional mouse model (41), but 1 PrP peptide detected in the brains of isotopically labeled mice showed a half-life of 5 days (42). The ASO used here reaches maximal activity at the RNA level within approximately 7 days (6), yet appeared to achieve deeper protein suppression in brain parenchyma at 4 weeks than at 2 weeks after dosing in this study, which would be more consistent with the higher estimate for PrP half-life. We previously observed that following a single ASO treatment in mice, it takes 3 weeks for neuropathological markers to diverge between treated and untreated cohorts. Levels of plasma neurofilament light, a marker of neuronal damage, continue to decline for 6 weeks after dosing (6). These kinetics are consistent with a lag between engagement of the RNA target, reduction of protein levels, and amelioration of the downstream disease process. Together, these findings may inform timing considerations for dosing of PrP-lowering therapies.

Finally, we observed that CSF PrP slightly underestimated the depth of parenchymal PrP knockdown at 4 weeks, which could reflect either a different half-life or different dose-response relationship for PrP released into CSF. More detailed pharmacodynamic modeling in multiple species will be required to link CSF PrP readouts in humans to estimates of brain parenchyma PrP reduction.

Our assay should serve as a tool for further development of PrP-lowering therapies, and our findings support the utility of PrP quantification as a tool in the development paths of such therapies.

## Methods

### Assay development.

Initial assay development was undertaken by BioAgilytix Boston (then known as Cambridge Biomedical). Antibody pair and other key assay configuration parameters were established, and the assay was subjected to a full validation study for rat CSF (Supplemental Appendix 3) compliant with World Health Organization Good Clinical Laboratory Practice Regulations (GCLP) (43). The assay was then transferred to the Broad Institute where the standard curve points and reagent concentrations were modified to yield the final assay conditions described below. Validation for mouse brain homogenate and all subsequent studies were performed at the Broad Institute.

### Cross-species PrP ELISA.

The exact assay protocol and checklist referred to at the bench while running the assay are provided as Supplemental Appendices 1 and 2. The method is briefly summarized as follows.

To prepare biotinylated detection antibody, 1 mg of EZ-Link Sulfo-NHS-SS-Biotin (Thermo Fisher Scientific A39258) was combined with 0.09 mg of 8H4 antibody (Abcam ab61409). Conjugated antibody was purified using Zeba spin desalting columns (Thermo Fisher Scientific 89889) and quantified by NanoDrop. Assay buffer of 0.05% wt/vol Tween (Teknova T0710) and 5% BSA in 1X CSHL PBS was 0.22 μm vacuum-filtered and stored at 4°C. Wash buffer was 0.1% Tween in 1X CSHL PBS, stored at room temperature.

Clear flat-bottom MaxiSorp plates (Thermo Fisher Scientific 439454) were coated overnight at 4°C with 2.0 μg/mL EP1802Y capture antibody (Abcam ab52604) in PBS, sealed with clear adhesive MicroAmp Film (Life 4306311) and then washed 3 times with 300 μL wash buffer and tapped dry (subsequent washes followed this same procedure). Plates were blocked with 250 μL assay buffer (0.05% Tween-20, 5% BSA, 1X PBS), sealed at room temperature for 1–3 hours and then washed. A fresh aliquot of recombinant PrP was thawed to make a new standard curve for every plate (5, 2, 0.8, 0.32, 0.13, 0.05, and 0 ng/mL). Standards, quality control samples (QCs), and samples were diluted into assay buffer in microcentrifuge tubes and 100 μL was added per well. Plates were sealed and incubated with sample for 60–75 minutes and then washed. Biotinylated 8H4 detection antibody was added at 0.25 μg/mL in 100 μL assay buffer; plates were sealed, incubated 60–75 minutes, and then washed. Pierce High Sensitivity Streptavidin-HRP (Thermo Fisher Scientific 21130) was added at 24.69 ng/mL in 100 μL assay buffer; plates were sealed, incubated for 30 minutes, and then washed. 100 μL TMB (Cell Signal 7004P4) was added; plates were incubated in darkness but monitored periodically for absorbance at 605 nm. After 30 minutes or when absorbance for the 5 ng/mL standard reached 0.8, whichever came sooner, 100 μL of stop solution (Cell Signal 7002L) was added; plates were shaken briefly and then read at 450 nm with 630 nm background subtraction on a Spectramax M5 plate reader (Molecular Devices). Standard curves were fit with a 4-point hill slope curve using the minpack.lm package (44) in R. FDA guidance (23) was followed for non-GLP validation of the assay in mouse brain homogenate (Supplemental Tables 1 and 2, and Supplemental Figures 1 and 2). To obtain diluted concentrations within the dynamic range of the assay, brains were run at a final dilution of 1:200 (for instance, 1:20 dilution of 10% wt/vol homogenate), and CSF samples were run at dilutions of either 1:20 (rat), or ≥1:40 (human; 5 samples at or near upper limit of quantification at 1:40 were re-run at 1:80).

For plates prepared in the prion laboratory, the protocol was modified as follows. All reagents, standard curves, and QCs were diluted to working concentrations in the morning before beginning the protocol and were kept at 4°C throughout the day. Instead of tapping dry, wells were washed with 190 μL of wash buffer using a multichannel pipette ejecting waste into a bleach bath. Plates were read at 450 nm with 620 nm background subtraction on a Fluostar Optima plate reader (BMG Labtech).

### Recombinant PrP.

Recombinant PrP was expressed in *E*. *coli* and purified from inclusion bodies as described previously (45) using a standard protocol (46). Recombinant protein preparations were quantified by amino acid analysis (New England Peptide), purity assessed by Coomassie staining (Supplemental Figure 3), and identity confirmed by LC/MS as described (45) (Supplemental Figure 3). All constructs were expressed in a pET-41a(+) vector. Human, mouse, Syrian hamster, and bank vole (M109) constructs were generous gifts from Byron Caughey, Andrew G. Hughson, and Lynne D. Raymond at Rocky Mountain Laboratories. Rat and cynomolgus macaque constructs were produced by GenScript. Sequences (Supplemental Table 3) were translated using ExPASy (47) and aligned using Clustal Omega (48, 49) (Supplemental Figure 3). Aliquots of 40 μL with 0.03% wt/vol CHAPS (MilliporeSigma C9426) were frozen at –80°C.

### MRM.

MRM was performed as described (12). For rat brain analysis, 1 hemisphere of cortex and subcortex (without cerebellum or brainstem) were homogenized at 20% wt/vol in cold 0.2% wt/vol CHAPS, 1X PBS, and 1 tablet protease inhibitor (Roche cOmplete, MilliporeSigma 4693159001) per 10 mL; then diluted to 0.5% wt/vol homogenates in artificial CSF, with final concentration of 0.03% wt/vol CHAPS. Rat brain and CSF were processed and analyzed in singlicate, with single-residue ^15^N/^13^C-labeled heavy peptides as the reference standard and light:heavy (L/H) peak area ratio to estimate the concentration of PrP in each sample.

All human CSF samples were analyzed in duplicate with fully ^15^N-labeled HuPrP23-231 as the reference standard and light:^15^N (L/^15^N) peak area ratio used to calculate PrP concentration. Test-retest analysis utilized CSF pairs taken 2–4 months apart from 5 individuals deliberately selected to include 2 individuals with, and 3 without, CSF processing anomalies, as this affects test-retest reliability for PrP (11). After confirmation of test-retest reliability (mean test-retest CV = 4.5% to 15.7% for the 4 peptides with technical replicate CV < 15%; Supplemental Table 4), we proceeded to analyze CSF samples from only the first study visits, rather than all study visits, for the remaining *n* = 42 study participants. For *n* = 29 replicates, the VVEQMCITQYER peptide was more abundant in met-ox than reduced form; for these replicates, the L/^15^N ratio was calculated using the met-ox form of both light and labeled protein. Any sample-peptide combination with technical replicate CV greater than 15% was excluded from downstream analysis.

### Tissue processing.

Brains for analysis were homogenized at 10% wt/vol (e.g., 100 mg tissue + 1 mL buffer), except where otherwise indicated, in cold 0.2% wt/vol CHAPS, 1X PBS, and 1 tablet protease inhibitor (Roche cOmplete, MilliporeSigma 4693159001) per 10 mL, using 3 × 40 second pulses on a Bertin MiniLys homogenizer in 7 mL tubes pre-loaded with zirconium oxide beads (Precellys KT039611307.7). Human CSF was collected as described (10, 11), and rat CSF collection is detailed below; 0.03% wt/vol CHAPS (final concentration) was added to all CSF samples at the earliest possible moment after collection.

### Patient samples.

CSF from asymptomatic *PRNP* mutation carriers and controls was collected through the Massachusetts General Hospital prion disease biomarker study and included samples previously described (11). Participants were recruited through PrionRegistry.org, Rally (Mass General Brigham), Prion Alliance, and CJD Foundation. Participants analyzed here had no mutation (*n* = 21), E200K (*n* = 12), D178N (*n* = 6), P102L (*n* = 4), or other *PRNP* mutation (*n* = 4), and each made 1–5 study visits (mean: 2.3) spanning a time period of up to approximately 3.5 years. Immediately after CSF sample collection, 0.03% CHAPS (final concentration) was added.

Postmortem human brain samples were obtained from the Massachusetts Alzheimer’s Disease Research Center (MADRC). Samples were from *n* = 5 control individuals without dementia, aged from approximately 50 to 90 years, postmortem intervals of 8–86 hours, *n* = 4 male and *n* = 1 female.

### Animals.

All mice were C57BL/6. PrP ZH3 knockout mice (50) on a C57BL/6J background were crossed to C57BL/6N animals. RML prions were intracerebrally inoculated at age 6–10 weeks as described (6). Intracerebroventricular (ICV) ASO injections in mice were as described (6) and were performed at age 7–10 weeks, except in prion-inoculated animals, where injections were at age 16 weeks (60 days after inoculation at 8 weeks of age). Mice for brain regional studies were 12–14 weeks old. For mouse brains, whole hemisphere analyses included cerebella but excluded brainstem and olfactory bulbs. Ipsilateral (right) hemispheres were used for RNA analysis and contralateral (left) for protein analysis.

Rats were Sprague-Dawley males (age study; age 3–11 months) and females (pharmacodynamic study; body weight ~300 g at study start). Rat CSF collection was performed under terminal anesthesia as follows. Occipital and nuchal areas were trimmed of hair and wiped with 70% ethanol. The heads of the rats were immobilized in a stereotaxic instrument (ASI SAS-4100) while being maintained on 3% isoflurane and warmed on a heating pad (Physitemp HP-1M). The nose was rotated down 45° and held in this position with the nose bar of the stereotax. A 90° hemostatic forceps (Roboz RS-7291) was depressed against the skin to locate the space between the trapezii and the base of the skull, and a 27G butterfly needle (MedVet International 26709) was held in a custom stereotaxic needle holder and attached to a 1 mL syringe, then inserted through the nuchal skin by lowering the dorsal/ventral knob of the stereotaxic instrument. The plunger of the syringe was withdrawn to create vacuum, and then the needle was lowered further, into the cisterna magna, until CSF began flowing into the butterfly tubing. When CSF flow ceased or blood was observed, the tubing was clamped with a hemostat and, if necessary, the tube was clipped at the meniscus of blood. The syringe was plunged to eject CSF into a low protein binding microcentrifuge tube (Eppendorf 022431081), and 3% wt/vol CHAPS stock solution was added at a 1:100 dilution to yield a final concentration of 0.03% CHAPS.

Cynomolgus macaque (*n* = 3 male, *n* = 3 female, age 2–4 years) tissue punches were obtained from tissue archived at –80°C from control animals treated with artificial CSF as part of previous ASO studies.

### Intracerebroventricular injections in rats.

Rats were shaved and maintained at 3% isoflurane while being warmed with a heating pad (Physitemp HP-1M). They were placed in a stereotaxic instrument (ASI Instruments, SAS-4100) with 27° atraumatic ear bars (ASI Instruments, EB-927), with the rat gas adapter riser set to –6 mm to set the lambda and bregma landmarks flat. The scalp was swabbed with betadine and ethanol and a 1.5 cm midline scalpel incision was made, centered between the nose and occipital ridge. Sterile cotton-tipped applicators were used to retract the subcutaneous and periosteal tissues. A sterile 1 mm × 33 mm drill bit (McMaster Carr, 5058N51) in a hanging-style handpiece (McMaster Carr, 4454A14) was positioned above the bregma in a stereotactic handpiece holder (ASI Instruments, DH-1000) and then moved 1 mm caudal and 1.5 mm lateral. A bore hole was drilled at low speed and then a gastight 1710 small RN syringe (Hamilton 81030) was lowered through the skull hole, 3.7 mm from the surface of the brain into the lateral ventricle; 30 μL of injection solution was then ejected gradually over 10 seconds, and the needle was retracted after 3 minutes. The incision was closed with 5-O monofilament suture (Ethilon 661G-RL), and rats recovered in their home cages.

### Statistics.

All analyses utilized custom scripts in R 4.0.4. All statistical tests and all confidence intervals were 2-sided, including those for ANOVA (Figures 1 and 3), Spearman correlation (Figure 2) Kolmogorov-Smirnov tests (Figures 2 and 3), regression models (Figures 2 and 4), and ANCOVA (Figure 4). *P* values were nominal except for GTEx analyses, which were Bonferroni-corrected for the number of tissues studied. *P* values less than 0.05 were considered significant. All code and all raw data, except for potentially sensitive patient data from the clinical cohort, are available in a public git repository and can be used to reproduce the analyses herein: https://github.com/ericminikel/cns_prp_quant (main branch, commit dd7c52f, release v1.0). Skyline files for mass spectrometry data have been uploaded to Panorama at https://panoramaweb.org/mortprpjci2202fz.url under ProteomeXchange submission PXD031432.

### Study approval.

Collection and analysis of human clinical samples were approved by the Partners Institutional Review Board (protocol #2017P000214). Animal studies were conducted under approved Institutional Animal Care and Use Committee protocols (Ionis Pharmaceuticals P-0273, Broad Institute 0162-05-17, and McLaughlin Research Institute 2020-DEC-75).

## Author contributions

EVM and SMV conceived and designed the experiments. MAM, HTZ, AGR, JEG, EK, JO, PMD, TRC, CM, SWA, BAT, AM, MRM, and DEC performed the experiments. EVM, SMV, HTZ, JL, HBK, JM, DEC, and SEA supervised the research. EVM performed statistical analyses and drafted the manuscript. All authors reviewed and approved the manuscript.

## Supplementary Material

Supplemental data

## Version 1 02/08/2022

In-Press Preview

## Version 2 03/22/2022

Electronic publication
