# Supplementary material for: Regional variability and genotypic and pharmacodynamic effects on PrP concentration in the CNS
Source: JCI Insight. 2022 Mar 22;7(6):e156532. doi: 10.1172/jci.insight.156532 (PMC8986079; doi:10.1172/jci.insight.156532)
Supplement: Supplemental data [file jciinsight-7-156532-s187.pdf]

SUPPLEMENTARY MATERIAL

Mortberg et al., PrP concentration in the central nervous system: regional variability, genotypic effects, and pharmacodynamic impact

Development and evaluation of the cross-species PrP ELISA assay.

Four commercially available antibodies with advertised species cross-reactivity were screened in all possible capture-detection configurations to identify suitable pairs for sandwich ELISA. This screen yielded four hits with promising signal-to-noise ratio (Figure S1A). All of these configurations proved dose-responsive and exhibited at least some cross-reactivity (Figure S1B-E). The EP1802Y capture and 8H4 detection configuration was selected as having the most similar dose-response curves for recombinant rat and human PrP (Figure S1E). An initial configuration of this assay was then validated for rat CSF (Appendix 3).

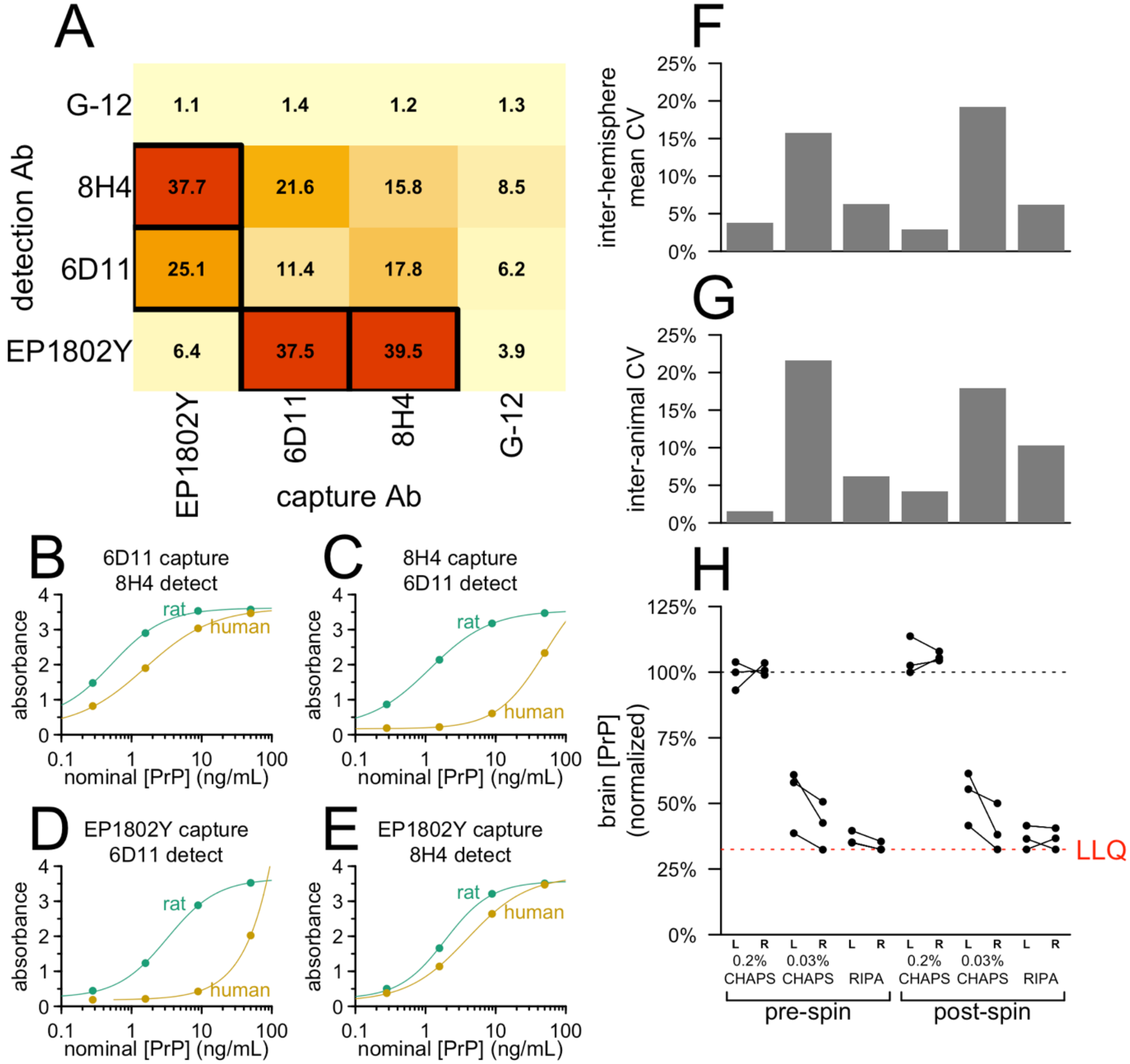

Figure S1. Development of the cross-species PrP ELISA. A) *Signal-to-noise ratios (450 nm absorbance for 20 ng/mL vs. 0 ng/mL recombinant rat PrP) for screened antibody pairs.* ***B-E)*** *Dose-response curves for recombinant human and rat PrP for top four antibody pairs.* ***F)*** *Mean*

CVs comparing right vs. left brain hemispheres of the same animal, ***G)*** *mean CVs between animals, and* ***H)*** *normalized response data for brains homogenized with the indicated detergents.*

PrP in CSF exhibits enormous inter-individual variability if preanalytical variables are not properly controlled (1), and we hypothesized the same might be true for PrP in brain tissue. We therefore sought to establish conditions for brain homogenization that would enable reliable PrP quantification. We hemisected frozen brains from wild-type mice, and for each animal, both right and left hemispheres were homogenized at 10% wt/vol in either 0.2% or 0.03% wt/vol CHAPS, or RIPA buffer (Pierce 89900, 25 mM Tris HCl pH 7.6, 150 mM NaCl, 1% NP-40, 1% sodium deoxycholate, 0.1% SDS). Homogenization in 0.2% CHAPS, just below the critical micelle concentration (2, 3), resulted in tight agreement of PrP concentration between hemispheres (mean CV = 3.8%, Figure S1F) and between animals (mean CV = 1.5%, Figure S1G), with >2x higher PrP recovery (Figure S1H) compared to 0.03% CHAPS or RIPA.

After establishing the final standard curve points and assay concentrations (see Methods and Appendices 1-2), we sought to characterize the assay's performance and determine whether it is fit for purpose for measuring PrP in mouse brain tissue in preclinical drug discovery experiments. We prepared quality control (QC) samples using mouse brain homogenized at 10% wt/vol in 0.2% CHAPS (Table S1), intended to represent brains with 100%, ~50%, 10%, and 0% wild-type levels of PrP (high, mid, low, and negative QCs respectively) and analyzed them at a final 1:200 dilution (1:20 dilution of 10% wt/vol homogenate). A non-GLP validation following FDA guidance (4) determined a dynamic range of 0.05 to 5 ng/mL, with acceptable precision for both calibrators and QCs across this range, except for the low QC sample, which had a high inter-plate CV (32.7%; Table S1). We further conducted a stability assessment for common preanalytical perturbations (Table S2). In contrast with CSF (1), brain homogenate did not disclose a decrease in PrP concentration upon transferring between plastic tubes (Table S2). Instead, the most important variable was time the brain homogenate spent at room temperature or 4°C, with apparent PrP concentration increasing by 29-56% after 4 hours at either temperature.

Table S1. Performance of calibration curve and quality control samples in cross-species PrP ELISA. *Inter-plate data are across seven validation plates; intra-plate data are from six replicates on one validation plate. For the analyses shown here, only standard curve points from 0.05 to 5.00 ng/mL were included in the four-point curve fit. *When the 0.02 ng/mL standard was included in the fit, its own mean backfit concentration was 0.01 ng/mL and its intra- and inter-plate CVs were 38.1% and 39.5% respectively.*

calibration curve						
	absorbance			fitted concentrations		
nominal concentration (ng/mL)	mean	CV	fold blank	mean	intra-plate CV	inter-plate CV
5.00	2.174	3.7%	59.8	5.00	7.1%	0.1%
2.00	1.318	3.4%	36.2	2.00	4.3%	0.2%
0.80	0.637	2.4%	17.5	0.80	2.5%	0.9%
0.32	0.280	4.5%	7.7	0.32	4.8%	2.6%
0.13	0.136	6.0%	3.7	0.13	7.5%	5.6%
0.05	0.076	3.8%	2.1	0.05	7.4%	14.0%
0.02	0.053	9.0%	1.5	—*	—*	—*
0.00	0.036	7.6%	1.0	—	—	—

quality control (QC) samples							
		absorbance		fitted concentrations			
name	composition	mean	CV	mean	% high QC	intra-plate CV	inter-plate CV
High QC	WT	0.502	5.7%	123.93	100.0%	5.9%	11.9%
Mid QC	het KO	0.265	4.3%	61.99	50.0%	4.5%	14.5%
Low QC	90% hom KO / 10% WT	0.099	3.5%	17.13	13.8%	4.3%	32.7%
Neg QC	hom KO	0.055	13.9%	5.40	—	—	—

Table S2. Stability assessment of mouse brain homogenate in cross-species ELISA. *The indicated (n) number of aliquots of the same high and low PrP brain homogenate samples were subjected to a battery of conditions to determine mean apparent PrP concentration, coefficient of variation (CV) and absolute relative error (%RE).*

condition	high PrP (WT brain)				low PrP (90% KO / 10% WT)			
	n	mean	CV	%RE	n	mean	CV	%RE
Freshly Thawed	8	110.7	6%	—	8	20.2	14%	—
Room Temp 4hrs	4	161.3	2%	**46%**	4	31.5	2%	**56%**
4°C 4hrs	4	142.3	1%	**29%**	4	26.1	10%	**29%**
Freeze Thaw 1 cycle	4	117.5	4%	6%	4	24.3	12%	20%
Freeze Thaw 2 cycles	4	129.0	4%	17%	4	29.3	9%	**45%**
Transfer Plastic 1 cycle	4	111.0	5%	0%	4	24.6	7%	22%
Transfer Plastic 2 cycles	4	117.3	6%	6%	4	25.3	8%	25%
Transfer Plastic 3 cycles	4	127.4	4%	15%	4	22.6	17%	12%

We sought to determine across what dilutions the assay might exhibit the property of parallelism, meaning that a sample plated at different dilutions results in the same dilution-adjusted concentration. The adjusted concentrations for all QCs rose at progressively weaker dilutions, even up to the lower limit of quantification of the assay (Figure S2A). However, the relative concentration of PrP in mid and low QC samples compared to the high QC remained constant regardless of dilution (Figure S2B). This suggested that while progressive dilution of brain homogenate into assay buffer changes the apparent concentration of PrP in this assay, progressive dilution of endogenous PrP into brain homogenate does not. This was confirmed by preparing a 7-point dilution series of wild-type brain into PrP knockout mouse brain, which resulted in a linear response at a 1:200 final dilution (Figure S2C). Thus, this assay exhibits a linear response to PrP concentration in brain tissue, provided that brain samples to be compared are plated at the same dilution into assay buffer. For three control human CSF samples, however, parallelism was observed over dilutions from 1:5 to 1:80 (Figure S2D), in agreement with findings from a commercial PrP ELISA kit (1). Standard curves of five species' recombinant PrP reacted identically in our assay, while a sixth species, Syrian hamster, exhibited ~3-fold lower, but still dose-responsive, reactivity (Figure S2E; see Figure S3 and Supplemental Discussion). For *N*=64 human CSF samples analyzed by both cross-species PrP ELISA and the commercially available BetaPrion ELISA kit, the rank order of concentrations was closely preserved (rho = 0.84, Spearman's correlation), while the absolute PrP concentration read out in cross-species PrP ELISA was ~6-fold lower (Figure 2F; see "Discussion of assay validation" status below).

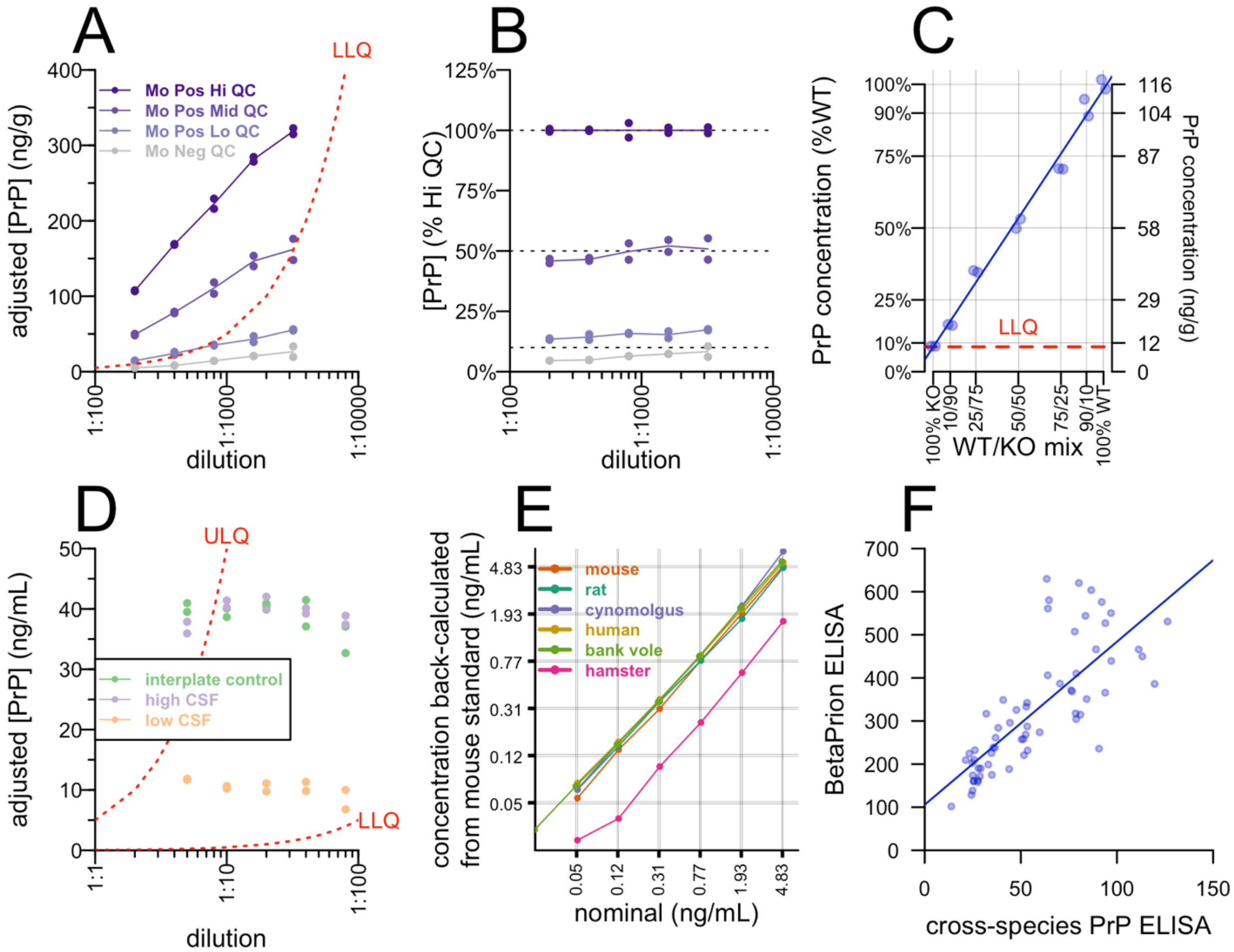

Figure S2. Parallelism, specificity, cross-reactivity, and comparison with BetaPrion ELISA. A) *QC samples were plated at dilutions from 1:200 to 1:1,600, the y axis indicates the apparent concentration after adjusting for dilution.* ***B)*** *The data from A normalized to the adjusted concentration of the high QC.* ***C)*** *Specificity assessed by a dilution series of wild-type into knockout brain homogenate. The blue line is the best fit.* ***D)*** *Control human CSF samples were plated at dilutions from 1:5 to 1:80, y axis indicates dilution-adjusted concentration as in A.* ***E)*** *AAA-quantified recombinant PrP from six species was plated at nominal concentrations indicated by the x axis, the y axis shows the apparent concentrations back-fit to the mouse standard curve.* ***F)*** *Best fit between cross-species PrP ELISA and BetaPrion ELISA for N=64 human CSF samples from N=29 individuals analyzed by both methods.*

Table S3. Recombinant PrP constructs. *Note that N-terminal methionines in E. coli are expected to be cleaved when followed by G but not when followed by K* (5)*, see Figure S3. The first K in each sequence corresponds to residue K23 in humans or its ortholog in other animals, the first residue after PrP's signal peptide.*

batch	species	identity	sequence
5	human	HuPrP23-231	MKKRPKPGGWNTGGSRYPGQGSPGGNRYPP<br>QGGGGWGQPHGGGWGQPHGGGWGQPHGG<br>GWGQPHGGGWGQGGGTHSQWNKPSKPKTN<br>MKHMAGAAAAGAVVGGLGGYMLGSAMSRPII<br>HFGSDYEDRYYRENMHRYPNQVYYRPMDEYS<br>NQNNFVHDCVNITIKQHTVTTTTKGENFTETDV<br>KMMERVVEQMCITQYERESQAYYQRGSS
16	mouse	MoPrP23-230	MKKRPKPGGWNTGGSRYPGQGSPGGNRYPP<br>QGGTWGQPHGGGWGQPHGGSWGQPHGGS<br>WGQPHGGGWGQGGGTHNQWNKPSKPKTNL<br>KHVAGAAAAGAVVGGLGGYMLGSAMSRPMIH<br>FGNDWEDRYYRENMYRYPNQVYYRPVDQYS<br>NQNNFVHDCVNITIKQHTVTTTTKGENFTETDV<br>KMMERVVEQMCVTQYQKESQAYYDGRRS
37	bank vole	BvPrP23-230	MKKRPKPGGWNTGGSRYPGQGSPGGNRYPP<br>QGGGTWGQPHGGGWGQPHGGGWGQPHGG<br>GWGQPHGGGWGQGGGTHNQWNKPSKPKTN<br>MKHVAGAAAAGAVVGGLGGYMLGSAMSRPMI<br>HFGNDWEDRYYRENMNRYPNQVYYRPVDQY<br>NNQNNFVHDCVNITIKQHTVTTTTKGENFTETD<br>VKMMERVVEQMCVTQYQKESQAYYEGRS
50	rat	RaPrP23-231	MKKRPKPGGWNTGGSRYPGQGSPGGNRYPP<br>QSGGTWGQPHGGGWGQPHGGGWGQPHGG<br>GWGQPHGGGWSQGGGTHNQWNKPSKPKTN<br>LKHVAGAAAAGAVVGGLGGYMLGSAMSRPML<br>HFGNDWEDRYYRENMYRYPNQVYYRPVDQY<br>SNQNNFVHDCVNITIKQHTVTTTTKGENFTETD<br>VKMMERVVEQMCVTQYQKESQAYYDGRRS
51	cynomolgus	CyPrP23-230	MKKRPKPGGWNTGGSRYPGQGSPGGNRYPP<br>QGGGGWGQPHGGGWGQPHGGGWGQPHGG<br>GWGQPHGGGWGQGGGTHNQWHKPSKPKTS<br>MKHMAGAAAAGAVVGGLGGYMLGSAMSRPLI<br>HFGNDYEDRYYRENMYRYPNQVYYRPVDQYS<br>NQNNFVHDCVNITIKQHTVTTTTKGENFTETDV<br>KMMERVVEQMCITQYEKESQAYYQRGS
71	Syrian hamster	SHaPrP23-232	MGKKRPKPGGWNTGGSRYPGQGSPGGNRYP<br>PQGGGTWGQPHGGGWGQPHGGGWGQPHG<br>GGWGQPHGGGWGQGGGTHNQWNKPSKPKT<br>NMKHMAGAAAAGAVVGGLGGYMLGSAMSRP<br>MMHFGNDWEDRYYRENMNRYPNQVYYRPVD<br>QYNNQNNFVHDCVNITIKQHTVTTTTKGENFTE<br>TDIKIMERVVEQMCTTQYQKESQAYYDGRRSS

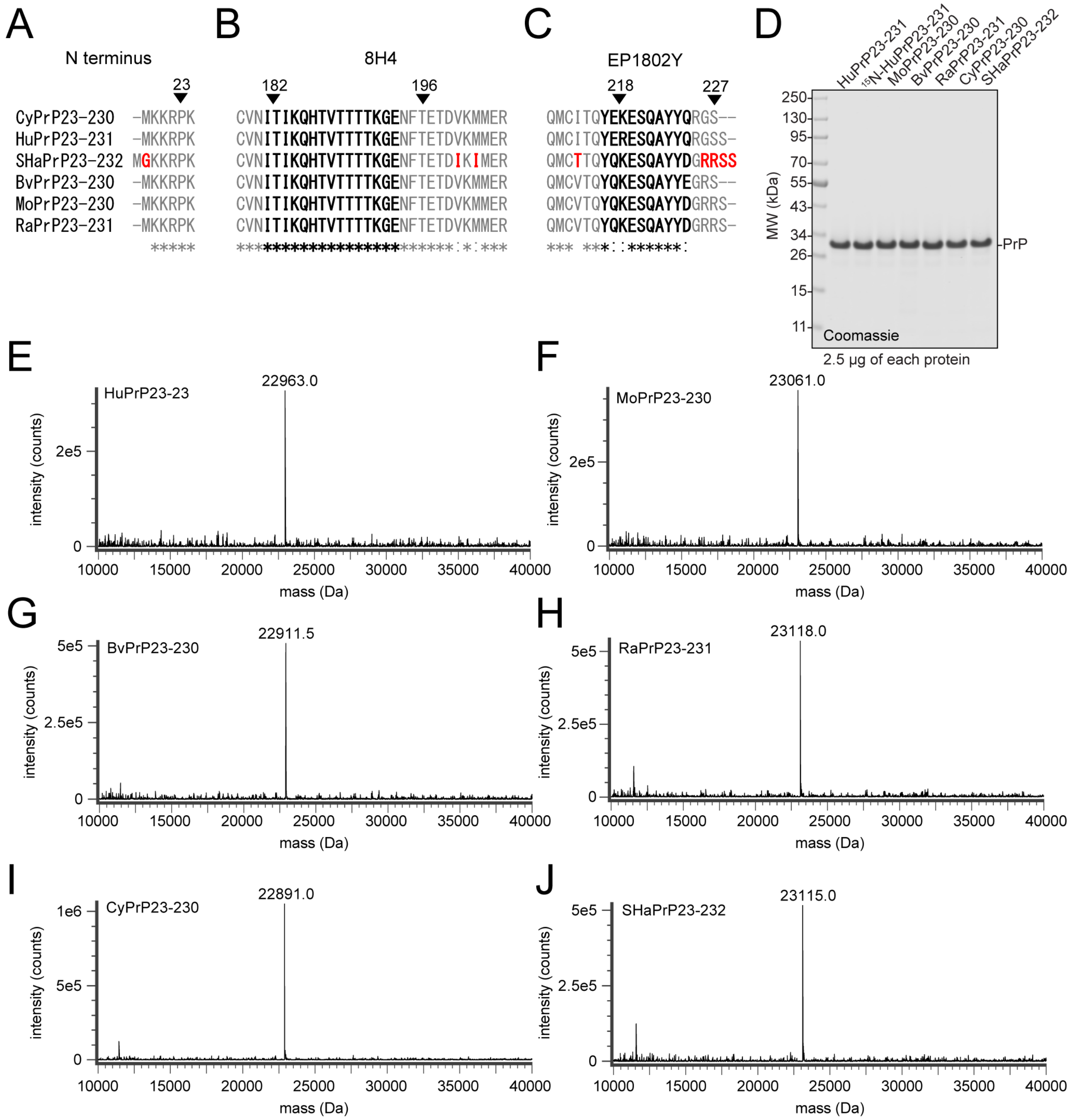

Figure S3. Epitope sequence, purity, and identity of recombinants. *A-C) Multiple alignment of vector sequences at the N and C termini and reported antibody epitopes* (6–8)*, translated using ExPASy* (9) *and aligned with Clustal Omega* (10, 11)*. Residues reported to be part of the 8H4 and EP1802Y epitopes are in bold, and residues unique to the Syrian hamster construct are highlighted in red. D) Coomassie-stained SDS-PAGE of the six recombinant batches used as standards in the ELISA assay, plus the* ^{15}*N-labeled HuPrP used as the standard in the MRM assay. E-J) Deconvoluted charge envelope of each recombinant standard run in intact protein LC-MS.*

There are several possible explanations for the reduced reactivity observed for Syrian hamster PrP. The N terminus of our other five constructs contain a retained N-terminal methionine (12), while the Syrian hamster construct contains a cleaved (5) N-terminal methionine followed by a

retained glycine (Figure S3A, red). The 8H4 antibody (6) has been found nonreactive for squirrel monkey PrP, which contains an I182V substitution (human codon numbering; CNVNVTIKQ), as well as for the human mutations H187R and E196K (7), suggesting its epitope spans from at least residue 182 to 196. These residues are invariant among the six species studied here (Figure S3B, bold). Syrian hamsters do harbor V203I and M205I substitutions (TETDIKIMERV) not found in any other species considered here (Figure S3B, red), though in order for these to affect 8H4 binding, the epitope would have to be discontinuous, as our MRM data indicate that our ELISA assay shows undiminished activity for PrP with the E200K mutation. Mutation scanning showed that the EP1802Y epitope was disrupted by mutations from residues 218 to 227 (human codon numbering) (8). Syrian hamster PrP in this span is identical to both rat and mouse PrP (Figure S3C, bold), however it does harbor a nearby I215T substitution not seen in any other species here (Figure S3C, red). Finally, our Syrian hamster construct contains one additional residue of C-terminal sequence present in the other species' genomes but not included in the recombinant constructs used here.

Although characteristics of this protein looked similar to the other batches employed here (Figure S3), we also considered technical explanations for the reduced reactivity of our Syrian hamster recombinant PrP. However, its elution curve was typical (Figure S4A), high purity by Coomassie (Figure S4B) was confirmed by size exclusion chromatography (Figure S4C), and identity was confirmed by LC/MS (Figure S4D). Despite all this, the lower reactivity compared to mouse PrP replicated identically across two plates (Figure S4E-F).

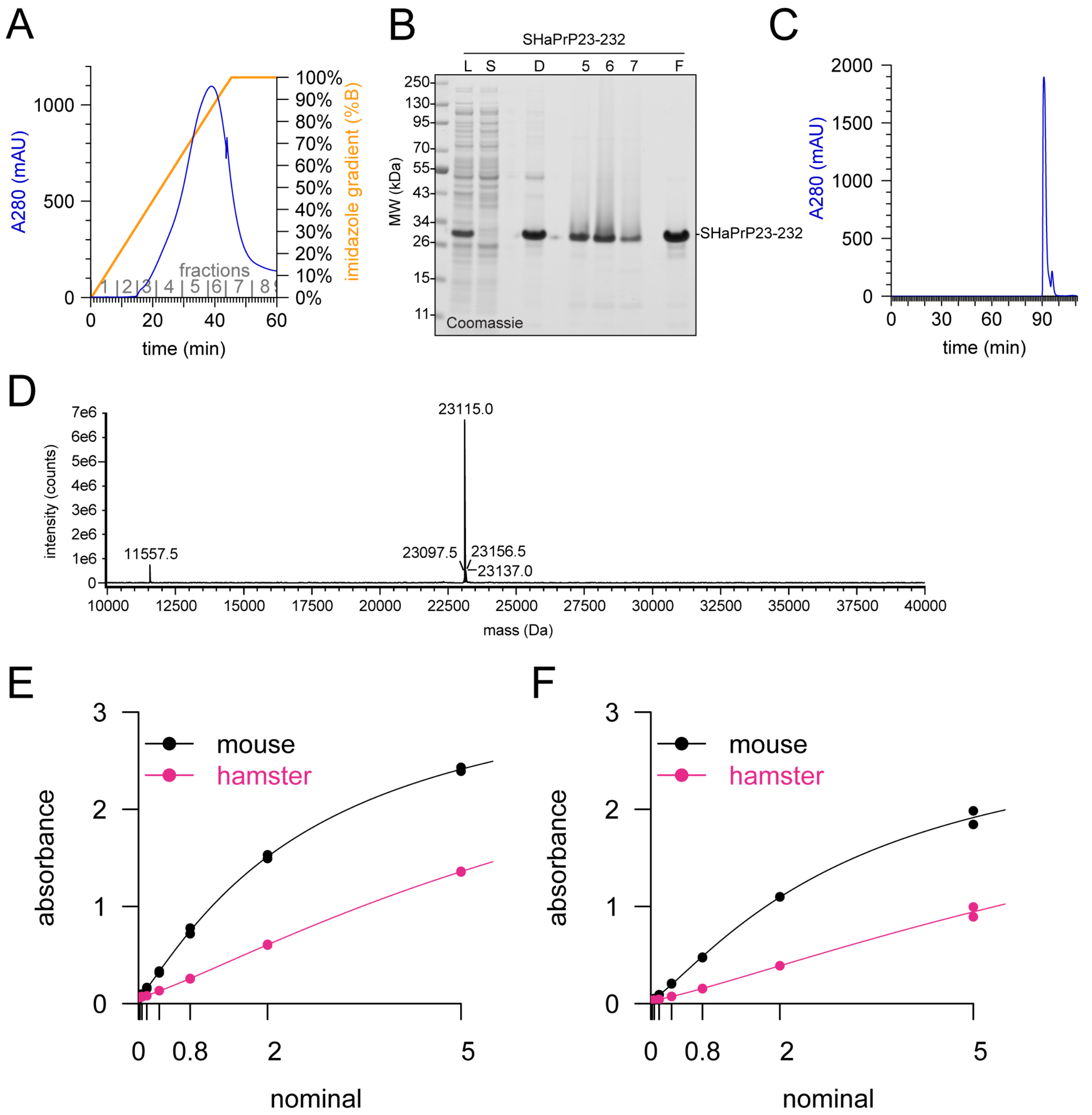

Figure S4. Hamster PrP purification and characterization. *Figure S2. SHaPrP23-232 purification and characterization. A) AKTA UV chromatogram of IMAC elution. B) Coomassie-stained SDS-PAGE of fractions from the purification of SHaPrP23-232. L, whole-cell lysate (diluted 1:20); S, soluble fraction (diluted 1:20); D, guanidinium denatured protein (diluted 1:20); 5-7, AKTA IMAC elution fractions; F, final SHaPrP23-232 sample used as an ELISA standard. C) SEC UV absorbance chromatogram. D) Deconvoluted charge envelope of SEC purified SHaPrP23-232 from intact protein LC-MS. The mass of 23115.0 Da corresponds to SHaPrP23-232 without the N-terminal methionine, and with the intramolecular disulfide bond in the oxidized state. E-F) Raw calibration curves for mouse and hamster PrP run on two separate ELISA plates.*

Discussion of assay validation status

Bioanalytical methods used in drug development should be "fit for purpose," with standards and expectations differing depending on the intended use case (4). The data presented here indicate that our cross-species PrP ELISA is suitable for quantifying target engagement of PrP-lowering therapeutics in mouse brain tissue, with certain caveats. Preanalytical variables — particularly time spent above freezing — must be properly controlled, samples are best compared at the same dilution, and inter-plate variability at the lower end of the dynamic range may be higher than desired, leading to a need for within-plate comparisons or additional technical replicates. PrP in brain homogenate, unlike CSF, does not appear highly sensitive to plastic exposure, perhaps because the high protein, lipid, and detergent content mitigate sticking. Surprisingly, for reasons not yet understood, measurable PrP in brain homogenate does appear to rise with increased time spent above freezing. Based on recombinant PrP binding curves, the assay appears applicable across at least six species of interest for prion research, although we did not perform full validation for all of them. Our data also support analysis of CSF in this assay, though we did not perform full validation in the final assay configuration for this matrix. Importantly, our assay uses a frozen recombinant PrP calibrator curve quantified by amino acid analysis (AAA). The one commercially available PrP ELISA, BetaPrion, uses lyophilized calibrators which appear to have PrP concentrations substantially lower than advertised (1), which limits that assay's capacity for absolute quantification of PrP (Dr. Ashutosh Rao, FDA, Oct 31, 2019). Our assay may be suitable for quantification of PrP in human CSF in a clinical trial setting, but because we are not a GLP laboratory, we did not pursue a formal validation for this use case. One important limitation is that the manufacturer (Abcam) recommends short-term storage at +4°C for the EP1802Y antibody, whereas long-term banking of a single lot of antibody at -80°C would be desirable for long-term analysis of clinical trial samples. We did not assess stability of either of our antibodies at -80°C. Finally, while we demonstrated target engagement of ASOs in prion-infected animals, we have not investigated whether our assay exhibits equal reactivity to PrP^{Sc} as it does to PrP^{C}. Some PrP antibodies, including 8H4, have been reported to exhibit diminished reactivity for PrP^{Sc} depending upon both the prion strain and the capture antibody employed (13).

Quality control of PrP MRM.

Among the five short-term test-retest CSF pairs analyzed, two peptides had high CVs (>30%), but these were peptides that also had high technical replicate CVs (>15%) among these samples (Table S4), perhaps because overall recovery (both of light and ^{15}N-labeled peptides) was relatively low. For the four peptides with low technical replicate CVs, test-retest CV was also low, supporting the analysis of just one CSF sample from each individual in Figure 3.

Table S4. Performance of peptides in MRM on human CSF. *For human sequence-matched peptides, we spiked fully ^{15}N-labeled protein and used L:^{15}N ratio as the assay readout. L:^{15}N mean value and technical replicate mean CV are for all human CSF samples analyzed; test-retest mean CV is for the five test-retest pairs analyzed.*

peptide	L:^{15}N mean value	L:^{15}N technical replicate mean CV	L:^{15}N test-retest mean CV
RPKPGGWNTGGSR	1.7	12.9%	15.7%
YPGQGSPGGNR	24.7	23.4%	38.2%
PIIHFGSDYEDR	16.4	9.0%	4.5%
GENFTETDVK	1.7	7.3%	7.1%
VVEQMCITQYER	17.9	6.9%	8.1%
ESQAYYQR	5.7	15.8%	34.5%

Common variants in *PRNP*.

We possessed only a small sample size of carefully handled CSF samples, and lacked genome-wide SNP data to control for population stratification. Nonetheless, in the interest of thoroughness, we chose to ask whether genotypes at two common *PRNP* variants with high prior probabilities for association with PrP expression showed any obvious correlation with CSF PrP concentration.

The coding variant rs1799990 (M129V) has dramatic effects on prion disease risk, duration, age of onset, clinical presentation, and histopathology across many subtypes of sporadic, acquired, and genetic prion disease (14). For example, the heterozygous genotype is strongly protective against sporadic CJD in a genotypic model (OR = 0.39, P = 1e-135) (15). It is the lead SNP for an eQTL for *PRNP* in several peripheral tissues but not in any brain region (Figure S5A). Our cohort contained only one VV individual, and there was no significant difference between CSF PrP in MM and MV individuals, whether all individuals or only mutation-negative controls were included (P = 0.06 or P=0.18, Kolmogorov-Smirnov test; Figure S5B).

Non-coding variant rs17327121, located 72 kb upstream of *PRNP*, is the lead SNP for an eQTL in cerebellum and cerebellar hemisphere, with no evidence of association with *PRNP* expression in any other brain region (Figure S5C). This SNP has not been reported to associate with prion disease risk, although neither it nor any SNP in tight linkage disequilibrium (r^2 > 0.5 in CEU, computed using LDlink (16)) was genotyped or imputed in the largest sporadic CJD GWAS to date. None of the pairwise differences in CSF PrP between genotypes were significant (P > 0.2 for all pairs, Kolmogorov-Smirnov test; Figure S4D).

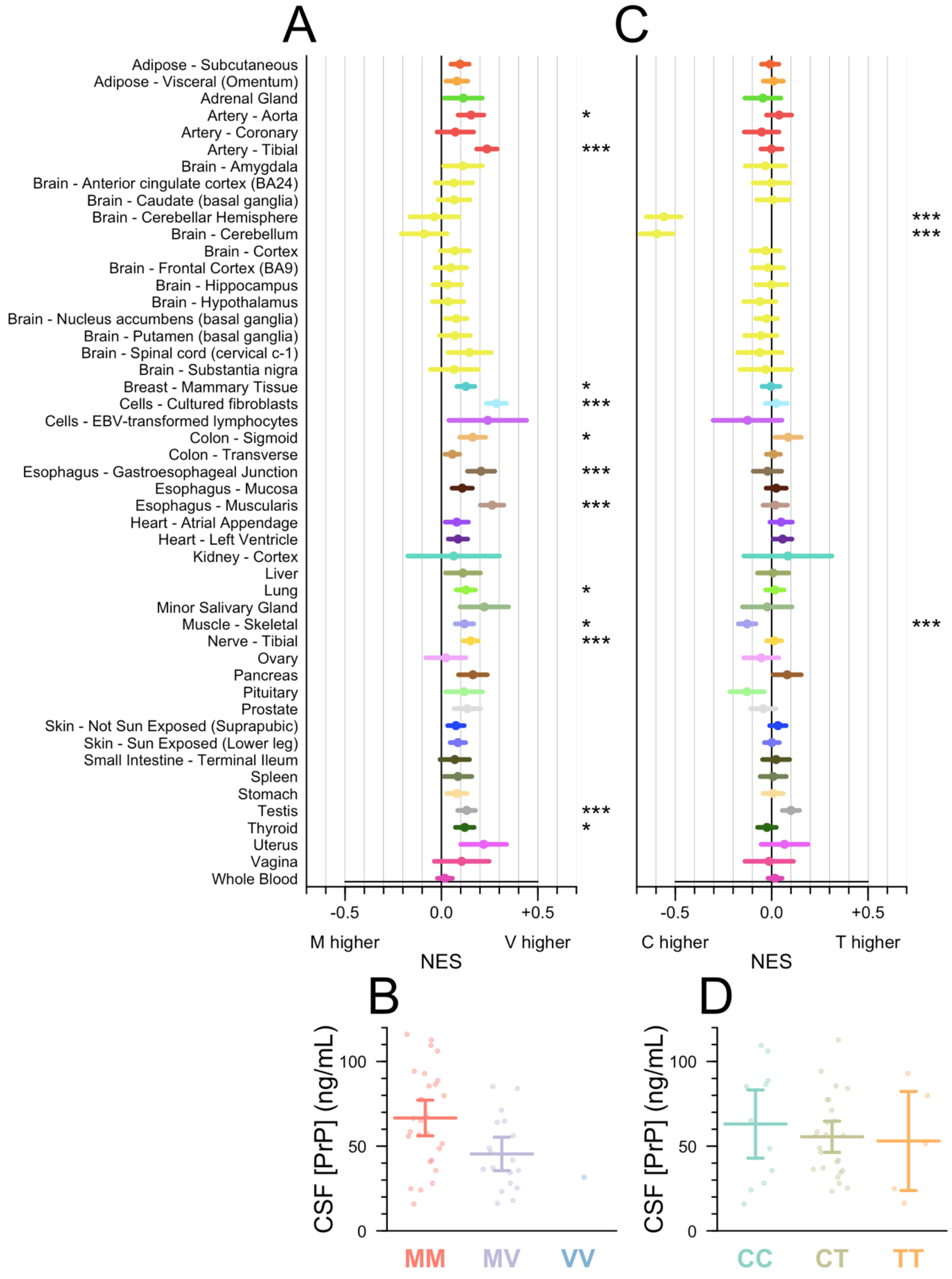

Figure S5. Common PRNP SNPs and CSF PrP. A) *PRNP multi-tissue eQTL data for rs1799990 reproduced from the GTEx browser (gtexportal.org). Positions to the right of the zero*

indicate that the 129V haplotype is associated with higher PRNP RNA expression in certain tissues than the reference 129M haplotype. The x axis is normalized effect size (NES), which is performed on normalized expression values with no direct biological interpretation (17). *Empirical thresholds for significance* (17) *in GTEx v8 vary by tissue down to 1e-5; symbols displayed here are as follows: * P < 1e-5, ** P < 1e-6, *** P < 1e-7. B) rs1799990 genotype and CSF PrP for all individuals in our MGH cohort. C) As panel A but for rs17327121. Positions to the left of the zero indicate that the reference allele, C, is associated with higher expression in cerebellum and cerebellar hemisphere than the alternate allele, T. D) rs17327121 genotype and CSF PrP in our MGH cohort.*

Supplementary References

1. Vallabh SM et al. Prion protein quantification in human cerebrospinal fluid as a tool for prion disease drug development. *Proc Natl Acad Sci U S A* 2019;201901947.

2. Hjelmeland LM. A nondenaturing zwitterionic detergent for membrane biochemistry: design and synthesis. *Proc Natl Acad Sci U S A* 1980;77(11):6368–6370.

3. Chattopadhyay A, Harikumar KG. Dependence of critical micelle concentration of a zwitterionic detergent on ionic strength: implications in receptor solubilization. *FEBS Lett* 1996;391(1–2):199–202.

4. U.S. Food and Drug Administration. Bioanalytical Method Validation. Guidance for Industry. [Internet]2018;https://www.fda.gov/downloads/drugs/guidances/ucm070107.pdf. cited March 8, 2019

5. Frottin F et al. The proteomics of N-terminal methionine cleavage. *Mol. Cell Proteomics* 2006;5(12):2336–2349.

6. Zanusso G et al. Prion protein expression in different species: analysis with a panel of new mAbs. *Proc Natl Acad Sci U S A* 1998;95(15):8812–8816.

7. Yin S et al. Human prion proteins with pathogenic mutations share common conformational changes resulting in enhanced binding to glycosaminoglycans. *Proc. Natl. Acad. Sci. U.S.A.* 2007;104(18):7546–7551.

8. Doolan KM, Colby DW. Conformation-dependent epitopes recognized by prion protein antibodies probed using mutational scanning and deep sequencing. *J Mol Biol* 2015;427(2):328–340.

9. Duvaud S et al. Expasy, the Swiss Bioinformatics Resource Portal, as designed by its users. *Nucleic Acids Res* 2021;49(W1):W216–W227.

10. Sievers F et al. Fast, scalable generation of high-quality protein multiple sequence alignments using Clustal Omega. *Mol Syst Biol* 2011;7:539.

11. Madeira F et al. The EMBL-EBI search and sequence analysis tools APIs in 2019. *Nucleic Acids Res* 2019;47(W1):W636–W641.

12. Minikel EV et al. Domain-specific quantification of prion protein in cerebrospinal fluid by targeted mass spectrometry. *Mol. Cell Proteomics* ; doi:10.1074/mcp.RA119.001702

13. Li R et al. Identification of an epitope in the C terminus of normal prion protein whose expression is modulated by binding events in the N terminus. *J Mol Biol* 2000;301(3):567–573.

14. Mead S, Lloyd S, Collinge J. Genetic Factors in Mammalian Prion Diseases. *Annu. Rev. Genet.* 2019;53:117–147.

15. Jones E et al. Identification of novel risk loci and causal insights for sporadic Creutzfeldt-Jakob disease: a genome-wide association study. *Lancet Neurol* 2020;19(10):840–848.

16. Machiela MJ, Chanock SJ. LDlink: a web-based application for exploring population-specific haplotype structure and linking correlated alleles of possible functional variants. *Bioinformatics* 2015;31(21):3555–3557.

17. GTEx Consortium. The GTEx Consortium atlas of genetic regulatory effects across human tissues. *Science* 2020;369(6509):1318–1330.

Appendix 1. Full assay protocol

Abbreviation	Name
Ab	Antibody
BSA	Bovine Serum Albumin
CHAPS	3-[(3-Cholamidopropyl)dimethylammonio]-1-propanesulfonate hydrate
CSF	Cerebrospinal fluid
CV	Coefficient of variation
HRP	Horseradish Peroxidase
LLQ	Lower Limit of Quantification
OD	Optical Density
PBS	Phosphate Buffered Saline
PrP	Prion Protein
QC	Quality Control
RE	Relative Error
rPrP	Recombinant Prion Protein
RT	Ambient Room Temperature
SD	Standard Deviation
TMB	3,3',5,5'-Tetramethylbenzidine
ULQ	Upper Limit of Quantification

Reagents

Name	Manufacturer	Catalog #
Zeba Spin Desalting Columns	Thermo Scientific	89889
EZ-Link Sulfo-NHS-SS-Biotin, No-Weigh format, 1 mg	Thermo Scientific	A39258
1X PBS	Broad Institute	N/A
Pierce BCA Protein Assay Kit	Thermo Scientific	23225
Anti PrP Ab 8H4	Abcam	ab61409

Equipment

Name	Manufacturer	Model #
37°C Incubator	Any	Any
NanoDrop	Thermo Scientific	NanoDrop 8000
SpectraMax M5 Plate Reader	Molecular Devices, Inc.	
Standard Orbital Shaker	VWR	1000

Biotinylation of 8H4 Antibody

Solution Preparations

1. Dilute 90 µg of 8H4 Ab (e.g. 50 µL of 1.8 mg/mL) with 1X PBS to bring to a total 200 µL.

Material Buffer Exchange

2. Remove the bottom closure on the Zeba column and place into a clean 15 mL conical tube. Keep the column upright and cap loosened.

3. Centrifuge the column device at 1000xG for 2 mins. Flow-through is discarded and the device was placed back into the same falcon tube.
4. 1 mL 1X PBS was added directly on top of the resin. The device is centrifuged at 1000 RCF for 2 mins and the flow-through was discarded. This step is repeated two more times for a total of 3 washes.
5. After the last wash step, the column is removed from the conical tube. Keeping the column upright, the bottom of the column is blotted off with a Kimwipe and is transferred to a clean 15 mL falcon tube.
6. 200 µL of 8H4 Ab is applied directly on top of the resin. After 1 min, 40 µL of 1X PBS is applied as a stacker.
7. The device is centrifuged at 1000xG for 2 mins. The column is discarded and the flow-through is kept on ice. The volume collected from the device is measured using a pipette and recorded.

Biotinylation

8. 180 µL of cold Milli-Q water is added into a microtube of 1 mg of NHS-SS-Biotin to prepare a 10mMol Biotin stock solution. The contents are mixed with a pipette and then mini-centrifuged to bring the solution down.
9. **See note for calculations** 14.6 µL of 10mM Biotin stock solution is added into the 8H4 Ab solution and mixed with a pipette.
10. The biotinylated 8H4 Ab solution is covered in foil and placed on the plate shaker for 30 mins at the setting “4” (~127 rpm).

Purification of Conjugated Protein

11. Remove the bottom closure on a new Zeba column and place into a clean 15 mL falcon tube. The column is kept upright and the cap loosened.
12. Following similar steps in the *Material Buffer Exchange* section, centrifuge the column device at 1000xG for 2 mins. The flow-through is discarded and the device was placed back into the same falcon tube.
13. 1 mL 1X PBS is added directly on top of the resin. The device is centrifuged at 1000xG for 2 mins and the flow-through was discarded. This step is repeated two more times for a total of 3 washes.
14. After the last wash step, the column is removed from the falcon tube. Keeping the column upright, the bottom of the column is blotted off with a Kimwipe and was transferred to a clean 15 mL falcon tube.
15. The biotinylated 8H4 Ab is applied directly on top of the resin. After 1 min, 40 µL of 1X PBS is applied as a stacker.
16. The device is centrifuged at 1000xG for 2 mins. The column is discarded and the flow-through is kept on ice.
17. The purified biotinylated 8H4 Ab solution is transferred into a clean 1.5 mL microtube, covered with foil and placed in the 4°C fridge. The final volume collected is measured using a pipette and recorded.

18. Use NanoDrop (Protein IgG concentration setting) to determine the concentration of the Ab. *Note: BCA can be used as an alternative to NanoDrop.*

****Note**

Calculations for Biotinylation

1. Calculate the concentration (mM) of the Sulfo-NHS-SS-Biotin to add to the reaction in order to obtain a specific molar excess. Typical challenge ratio is 20 Biotin: 1 molecule of protein

for a 20 molar excess. The 8H4 Ab has a concentration of 1.8mg/mL in 50µL solution. Antibodies in general are ~150 kDa or 150,000 mg/mmol.

Equation used:

$$Vol\,Ab \times Conc.Ab \times molar\,wt.Ab \times \frac{molar\,excess\,biotin}{moles\,of\,protein} = mmol\,Biotin$$

$$0.05\,mL \times \frac{1.8\,mg}{1\,mL} \times \frac{1\,mmol}{150{,}000\,mg} \times \frac{20\,mmol\,Bi}{1\,mmol} = 0.000012\,mmol\,Bi$$

2. To calculate the volume (in µL) of 10 mM Sulfo-NHS-SS-Biotin to add to the labeling reaction, where MW Biotin = 906.7 mg/mmol:

$$0.000012\,mmol\,Bi \times \frac{606.7mg}{1\,mmol} = 0.0072804\,mg\,Bi$$

$$0.0072804\,mg\,Bi \times \frac{1mL}{0.5mg} \times \frac{1000uL}{1mL} = \mathbf{14.6uL}\ of\ 10mM\ Biotin\ stock\ solution$$

Cross-Species PrP ELISA

Critical Equipment

Description	Manufacturer	Model Number	Broad ID
SpectraMax M5 Plate Reader	Molecular Devices, Inc.		101058

Critical materials, reagents, and supplies

Name	Manufacturer	Model #
Anti-PrP Ab EP1802Y	Abcam	ab52604
Biotin-8H4 detection antibody	Broad Institute	N/A
Recombinant mouse prion protein	Broad Institute	Mo PrP16

General materials, reagents, and supplies

Name	Manufacturer	Catalog #
TMB substrate	Cell Signaling Technology	7004P4
Stop solution	Cell Signaling Technology	7002L
CHAPS hydrate	Sigma	C9426
Milli-Q water	Millipore	N/A
Pierce High Sensitivity Streptavidin-HRP	Thermo Scientific	21130
96 Well Flat- Bottom Immuno Plate, MaxiSorp	Thermo Fisher Scientific	439454
0.22µm vacuum filter system	Corning	CLS431098
Bovine Serum Albumin	SeraCare Life Sciences	19K15A0018
1X PBS CSHL, pH 7.4	Broad Institute SQM	N/A
10% Tween-20 solution	Teknova	T0710
Seal, Clear Adhesive MicroAmp Film	Life Technologies	4306311

Reagent Preparation

- Wash buffer: 1X PBS with 0.1% Tween-20
  Dilute 10% Tween-20 to 0.1% in 1X PBS. Example: 990mL 1X PBS + 10mL 10% Tween-20. Store at RT for up to 2 months

- Assay buffer: 1X PBS with 5% BSA and 0.05% Tween-20
  Dilute the required amount of BSA and 10% Tween-20 in 1X PBS. Mix thoroughly. Example: 25 g BSA + ~400mL 1X PBS + 2.5 mL 10% Tween-20. Add 1X PBS to a final volume of 500mL. Filter through a 0.22 µm vacuum filter. Store at 4°C for up to 1 month.

- Standards
  Prepare high standard (Std01) by diluting stock MoPrP16 to 5ng/mL is assay buffer. Make 6 serial dilutions to produce the concentrations 2, 0.8, 0.32, 0.128, 0.0512, and 0.02048 ng/mL (Std02-07).
  The low standard (Std08) is neat assay buffer.
  Make a standard curve fresh from frozen, undiluted rPrP stock every time.

- QC Samples

The QC samples used are: Mo Pos Hi QC, Mo Pos Mid QC, Mo Pos Lo QC, and Mo Neg QC. The QCs are stored at -80°C and are in 40 µL aliquots.

Procedure

1. Prepare capture Ab solution by diluting capture antibody EP1802Y to 2.0 µg/mL in PBS. Vortex briefly to mix. Prepare enough Capture Ab solution to add 100 µL to each plate well plus a 10% excess. Seal the plate and store overnight at 4°C.
2. Wash plate 3x with 300 µL Wash buffer per well. Tap dry.
3. Block plate by adding 250 µL Assay buffer per well. Seal and incubate at RT for 1-3 hours.
4. Wash plate 3x with 300 µL Wash buffer per well. Tap dry.
5. While the plate is blocking, dilute standards, QCs, and samples in assay buffer and add 100 µL of each to the plate per plate map. Pipette up and down to mix. Seal and incubate at RT for 60-75 minutes.
6. Wash plate 3x with 300 µL Wash buffer per well. Tap dry.
7. Prepare detection Ab solution by diluting biotin-labeled 8H4 detection antibody to 0.25 µg/mL in assay buffer. Vortex briefly to mix. Prepare enough detection Ab solution to add 100 µL to each plate well plus a 10% excess. Seal the plate and incubate at RT for 60-75 minutes.
8. Wash plate 3x with 300 µL Wash buffer per well.
9. Prepare streptavidin-HRP solution by diluting streptavidin-HRP to 24.69 ng/mL in assay buffer. Vortex briefly to mix. Prepare enough Streptavidin-HRP solution to add 100 µL to each plate well plus a 10% excess. Seal and incubate at RT for 20-30 minutes. *(**Note: full 30 minutes recommended, otherwise the plate may not reach ~0.8 OD in the 30-minute time from during the TMB incubation step.)*
10. Wash plate 3x with 300 µL Wash buffer per well
11. Add 100 µL per well of TMB to plate. TMB solution should come to RT before using. Cover and incubate at RT until Std01 (5ng/mL) reaches ~0.8 OD. Pre-read plate at 605nm. If Std01 does not reach this OD within 30 minutes stop plate and read.
12. Add 100 µL per well of Stop solution to plate. Stop solution should come to RT before using. Mix well on plate reader briefly and read at 450nm and 630nm.

Appendix 2. ELISA working checklist

Day 1

1. Incubate the plate with 100 µL/well of **2 µg/mL EP1802Y Ab**. Seal and store at 4°C overnight.

Day 2

1. Wash plate 3X with 300 µL/well of **wash buffer** and tap dry
2. Block by adding 250 µL/well of **assay buffer** to plate. Seal and incubate at RT for 1-3 hr on benchtop

   Start time: __________

   Sealed: __________ → Stop time: __________
3. Prepare fresh standards from an aliquot of stock rPrP
4. Wash plate 3X with 300 µL/well of **wash buffer** and tap dry
5. Add 100 µL/well of **rPrP standards, mouse QCs, and samples** in duplicate. Seal and incubate at RT for 60-75 min.

   Start time: __________

   Sealed: __________ → Stop time: __________
6. Wash plate 3X with 300 µL/well of **wash buffer** and tap dry
7. Add 100 µL/well of **0.25 µg/mL biotin-8H4 Ab solution**. Seal and incubate at RT for 60-75 mins.

   Start time: __________

   Sealed: __________ → Stop time: __________
8. Wash plate 3X with 300 µL/well of **wash buffer** and tap dry
9. Add 100 µL/well of **24.69ng/mL streptavidin-HRP solution**. Seal and incubate at RT for 30 mins.

   Start time: __________

   Sealed: __________ → Stop time: __________
10. Wash plate 3X with 300 µL/well of **wash buffer** and tap dry.
11. Add 100 µL/well of RT **TMB**. Cover and incubate at RT on benchtop until Std. 1 (5ng/mL) reaches ~0.8 OD (pre-read at 605 nm) or 30 minutes max.

    Start time: __________

    Covered: __________ → Stop time: __________
12. Add 100 µL/well of RT **Stop Solution**. Mix well on plate reader briefly and read at 450 nm and 630 nm.

Appendix 3. GCLP validation results for rat CSF

Note: This validation study was performed by Bioagilytix Boston prior to the assay being transferred to the Broad Institute. The streptavidin-HRP concentration and the recombinant PrP standard curve points differ from the final assay configuration used at the Broad Institute. The results summary is shown below; the SOP and full validation report are available in this study's online GitHub repository.

Parameter	Expectation	Observed Performance
Intra-Assay Precision	CV ≤ 20% at VS-H, VS-M, VS-L	VS-H: 3.20%<br>VS-M: 5.12%<br>VS-L: 3.42%
Inter-Assay Precision	CV ≤ 30% at VS-H, VS-M, VS-L	VS-H: 12.4%<br>VS-M: 16.6%<br>VS-L: 21.3%
Accuracy	± 3 SD from average back calculated result of VS-H, VS-M, VS-L	VS-H: 13.0 - 28.4 ng/mL<br>VS-M: 4.00 – 11.9 ng/mL<br>VS-L: 1.61 – 7.28 ng/mL
Limits of Quantitation	Lowest and highest standards with CV and RE ≤ 25%	Std01: CV = 0.27%, RE = 0.181%<br>Std07: CV = 9.62%, RE = 3.35%
Parallelism	CV ≤ 30% for all concentrations within the LOQ	Run05 (64 to 128-fold): 26.2%<br>Run06 (8 to 64-fold): 16.9%<br>Run07 (8 to 16-fold): 27.3%
4°C Stability	RE ≤ 20% for at least 2/3 of aliquots per lot of CSF tested	100% of aliquots pass, CV of -5.9 to 6.7%
RT Stability	RE ≤ 20% for at least 2/3 of aliquots per lot of CSF tested	67% of aliquots pass, CV of -6.7 to -5.9%. Failing aliquot CV = -29.4%
Freeze Thaw Stability	RE ≤ 20% for at least 2/3 of aliquots per lot of CSF tested	100% of aliquots pass, CV of 0 to 6.7%
